# Frequency of depression and anxiety in patients with inborn errors of immunity: a cross-sectional study

**DOI:** 10.1186/s12888-026-08654-1

**Published:** 2026-09-15

**Authors:** Luca J. Wolff, Torsten Witte, Kai G. Kahl, Anna Meinecke, Gerrit Ahrenstorf, Georgios Sogkas, Stefanie Hillmer, Thea Thiele

**Affiliations:** 1 Department of Rheumatology and Immunology, Hannover Medical School, Hanover, Germany; 2 Department of Psychiatry, Social Psychiatry and Psychotherapy, Hannover Medical School, Hanover, Germany

**Keywords:** Inborn errors of immunity, Mental health, Depression, Anxiety, Self-isolation, Quality of life

## Abstract

**Background:**

Inborn errors of immunity are chronic, genetically caused disorders that impair immune function and often increase susceptibility to infection. Many patients self-isolate because of infection-related concerns, which may increase the risk of mental health problems. This study evaluated symptoms of depression and anxiety and the need to address mental health in primary care.

**Methods:**

In this cross-sectional study in Germany, fatigue, pain and quality of life were assessed using visual analogue scales. Routine clinical data including diagnosis, psychiatric comorbidities and susceptibility to infections were collected. Symptoms of depression and anxiety were evaluated using the PHQ-9 and GAD-7 questionnaires. Statistical analyses were performed to examine differences in self-isolation frequency and its association with pre-existing mental illness.

**Results:**

A total of 143 patients (88 women and 55 men, mean age 45.4 years, range 21–85) were included. The median PHQ-9 score was 5 (IQR 8) and the median GAD-7 score was 5 (IQR 7). Eleven of 140 patients with PHQ-9 scores of at least 10 had no psychiatric diagnosis. Overall, 26% of patients with relevant symptoms had not received a diagnosis or treatment. Patients who self-isolated because of infection concerns had significantly higher PHQ-9 (Spearman’s ρ = 0.52, *p* < 0.001) and GAD-7 scores (Spearman’s ρ = 0.48, *p* < 0.001).

**Conclusions:**

Self-isolation was associated with more severe symptoms of depression and anxiety, including in patients without known psychiatric disease. These findings highlight the need for greater awareness of mental health issues and multidisciplinary care in patients with inborn errors of immunity.

**Supplementary Information:**

The online version contains supplementary material available at https://doi.org/10.1186/s12888-026-08654-1.

## Introduction

Inborn errors of immunity (IEI) are a group of chronic, genetically caused diseases that affect the innate immune system. They are characterized not only by a higher susceptibility to infections, including persistent, severe and opportunistic infections, but also by autoimmune diseases due to immune dysregulation [1]. Additionally, IEI are associated with an increased risk of developing malignancies, particularly lymphoid and virus-associated malignancies [2]. The treatment varies widely depending on the specific IEI and its main clinical features. The options range from infection prophylaxis involving antibiotics, vaccinations and frequent inhalations to subcutaneous or intravenous Immunoglobulin G (IgG) replacement therapy and even bone marrow transplants in severe cases [3]. In patients with signs of autoimmune diseases due to immune dysregulation, immunomodulatory therapy is often necessary. The rising numbers of genetic variants identified in patients with IEI has enabled the development of more precise treatment strategies for a limited number of cases [4, 5].

IEI are not only characterized by susceptibility to recurrent or severe infections, but also by different degrees of immune dysregulation, autoimmunity, and chronic or recurrent inflammation [6]. These disease-related factors may be responsible for mental distress through two pathways. First, heterogenous disease manifestations, the unpredictable disease course and the need for long-term therapy and sometimes hospitalization represent psychological stressors [7]. Second, persistent or recurrent immune activation, as present in some subtypes of IEI, may influence the central nervous system through neuroimmune signalling and thereby contribute to fatigue, sleep disturbances and mood changes [8, 9]. For example, higher proinflammatory cytokines and chemokine levels can be observed in people suffering from depression [10]. These aspects of IEI all contribute to patients experiencing a reduced quality of life and sometimes patients even restrain themselves from human contacts to reduce the burden of recurrent or prolonged infections [11, 12].

According to the nationally representative DEGS1-MH study (German Health Interview and Examination Survey for Adults, Mental Health Module), the 12-month prevalence of any mental disorder in German adults is 27.7%. The most frequent mental disorders are anxiety (15.3%), mood (9.3%) and substance use disorders (5.7%), with higher rates in women, younger age groups, patients living without a partner, and low socio-economic status. Overall, 25% of all adults in Germany showed symptoms of depression or an anxiety disorder, while 11% of all adults showed symptoms of both [13].

Mental health disorders, particularly depression and anxiety, are significant comorbidities among patients suffering from chronic conditions such as inflammatory rheumatic diseases, diabetes or asthma [14–16]. In 2024, a Turkish study determined 44.2% and 61.5% of IEI patients being at risk for anxiety or depression, respectively [17]. Another study estimated that one third of the patients diagnosed with common variable immunodeficiency (CVID) were at risk for anxiety or depression and even two thirds of the female patients [18].

Previous studies have demonstrated increased mental health related symptoms, such as depression, anxiety, psychosocial burden, fatigue and reduced quality of life in patients with IEI [7, 19]. However, the psychosocial consequences of isolation have received little attention. Moreover, evidence from Germany is rare.

We hypothesized that concerns about infection risk may lead patients with IEI to limit their social interactions and become more socially isolated than healthy people. Thereby people with IEI are at risk to increase their vulnerability to depressive and anxiety-related symptoms. We further hypothesized that clinically relevant symptoms would be more common than formally diagnosed depressive or anxiety disorders, suggesting a substantial proportion of affected patients may remain unrecognized.

Accordingly, the primary objective of this study was to characterize the burden of depressive and anxiety symptoms in this cohort and to examine an association with self-reported isolation due to concerns about infection. We further hypothesized that a relevant proportion of patients with potentially clinically relevant depressive and anxiety symptoms as indicated by PHQ-9 or GAD-7 scores would have no previously documented psychiatric diagnosis. In addition, we performed exploratory analyses to investigate potential associations of depressive and anxiety symptom burden with clinical and patient-reported measures, including perceived infection susceptibility, pain, fatigue, quality of life, sleep disturbance and pre-existing psychiatric comorbidity.

In this study, we aimed to assess both the frequency of these symptoms in our cohort and the proportion of patients who, despite experiencing clinically relevant symptom severity, have not received a formal diagnosis of depression or an anxiety disorder. In doing so, we aim to characterise the burden of depressive and anxiety-related symptoms in our cohort and to explore whether these findings warrant further investigation of systematic symptom assessment in clinical practice.

## Methods

This monocentric, cross-sectional, observational study was conducted among patients visiting our outpatient clinic for inborn errors of immunity. Data were collected during routine clinical visits.

### Patient cohort and acquisition of clinical data

All adult patients diagnosed with inborn errors of immunity and presenting at our immunology outpatient clinic in Hannover, Germany between July 2024 and February 2025 were eligible for inclusion. Patients were included if they were able to complete patient-reported outcome measures and gave written informed consent. A total of 150 patients visiting our outpatient clinic were recruited. Patients were asked to rate their fatigue and pain levels, as well as their quality of life on a visual analogue scale (VAS) [20–22]. In addition to routine data such as diagnosis, psychiatric comorbidities, current susceptibility to infection, sleep disturbances and sleep medication use, depression and anxiety symptom questionnaires were used. To screen for depression, the Patient Health Questionnaire-9 (PHQ-9) was used with a cut-off of ten points to detect possible major depression [23]. The seven-item Generalized Anxiety Disorder Scale (GAD-7) was used with a cut-off of ten points to detect possible major anxiety disorder [24].

The study was conducted in accordance with the Declaration of Helsinki. This data analysis was approved by the Local Ethics Committee of Hannover Medical School (Approval Number: 11404_BO_S_2024).

### Patient and public involvement

Patients were informed about the study using a written leaflet; individual questions regarding the study protocol and participation were discussed orally, then the patients provided written consent. Questionnaires were filled during the routine follow-up appointments in our outpatient clinic. Neither patients nor the public were involved in designing the study itself.

### Assessment

Information including patients’ age, sex and diagnosis were obtained from the patients’ chart. Patients were asked about the onset and diagnosis of their IEI, any previously diagnosed psychiatric diseases, their therapy, and sleep disturbances (frequency, medications) using a separate questionnaire. The patients were also asked if their subjective susceptibility to infection was well controlled and if they had undergone social isolation due to concerns about infection. Social isolation was categorized according to its frequency (never, occasionally, frequently). Binary predictors were coded as 0 (absent/false) and 1 (present/true) with the category coded as 0 serving as the reference category unless stated otherwise. The binary variables included patients’ and physicians’ assessments of susceptibility to infections, sleep disturbances and diagnosed psychiatric disorders. In addition, patients provided a questionnaire including a visual analogue scale ranging from 0 to 10 for pain, fatigue and quality of life (QOL) (0 means no pain or fatigue/ best imaginable QOL and 10 worst imaginable pain or fatigue/ worst imaginable QOL). The patients also completed a Patient Health Questionnaire-9 (PHQ-9) to assess depressive symptoms. The questionnaire consists of nine items, each rated on a 4-point Likert scale ranging from 0 (“not at all”) to 3 (“nearly every day”), resulting in a total score between 0 and 27. Higher total scores reflect greater severity of depressive symptomatology. Established cut-off scores indicate mild (≥ 5), moderate (≥ 10), moderately severe (≥ 15) and severe (≥ 20) levels of depression [25]. In our dataset, the PHQ-9 demonstrated good internal consistency (Cronbach’s α = 0.89).

Anxiety symptoms were measured using the Generalized Anxiety Disorder-7 (GAD-7) scale. This seven-item instrument uses the same 4-point Likert scale as the PHQ-9, resulting in a total score ranging from 0 to 21. Commonly applied cut-off scores correspond to mild (≥ 5), moderate (≥ 10), and severe anxiety symptoms (≥ 15) [26]. In our cohort, the GAD-7 exhibited excellent internal consistency (Cronbach’s α = 0.91).

Patients who did not fully complete the PHQ-9 or GAD-7 questionnaire were excluded from this analysis.

### Methodology

Data were assessed for normality using histograms and the Shapiro–Wilk test. In cases when a normal distribution was confirmed, comparisons between two groups were assessed using independent t-tests. For non-normality distributed data, comparisons between two independent groups were conducted using the Wilcoxon–Mann–Whitney test, while comparisons among more than two groups were carried out using the Kruskal–Wallis test. Post-hoc analyses were performed using the Dunn–Bonferroni correction. Associations between ordinal or continuous variables were tested with Spearman’s rank correlation coefficient. Associations between categorial variables were analysed using the Chi-square test (χ²) or Fisher’s Exact test when expected cell counts were less than five. Reliability of patient-reported outcome measures was assessed via Cronbach’s alpha (α) coefficient. Inter-rater agreement between patients’ and physicians’ binary categorial assessments (“yes” or “no”) was evaluated with unweighted Cohen’s κ based on a 2 × 2 contingency table. Regression analysis was performed by fitting a least square regression model to the data. For multiple regression analysis after fitting a least square regression model to the data, an explanatory stepwise linear regression analysis was performed using backward stepwise elimination, with the minimum Bayesian information criterion (BIC) as the stopping rule. A second least-squares model was then fitted after excluding the previous eliminated predictors as a sensitivity analysis. Complete-case analysis was applied: Participants with missing values for any predictor were excluded from the respective model. Multicollinearity was assessed using variance inflation factors, and residual independence was assessed using the Durbin–Watson statistic. Because the analyses were exploratory, no formal sample-size or power-calculation was performed. All statistical tests were two-tailed and a p-value < 0.05 was considered statistically significant. Data were analysed using JMP 19 Student Edition. Figures were generated using JMP 19 Student Edition and Inkscape.

### Declaration of generative AI and AI-assisted technologies in the manuscript preparation process

During the preparation of this manuscript the authors used DeepL to translate parts of the paper and ChatGPT (Open AI) to edit text for grammar, spelling, organization and readability. After using these tools, the authors reviewed and edited the content as needed and take full responsibility for the content of the published article.

## Results

### Characteristics of the study population

A total of 150 patients were recruited of which four were excluded because they did not complete the questionnaires for PHQ-9 or GAD-7. A further three patients were excluded from the study, who did not meet the diagnostic criteria for an inborn error of immunity. In total, 143 patients with an inborn error of immunity were included, most of whom had common variable immunodeficiency (CVID) (Table 1). Baseline characteristics were largely comparable across the different groups of IEI. Although the cohort was predominantly composed of patients with IEI, 5 patients classified as secondary antibody deficiency were also included. Post-hoc sensitivity analyses excluding those five patients yielded results comparable to those of the primary analysis. The direction and magnitude of the associations, was well as the statistical conclusions remain unchanged. The only notable differences concerned sex distribution: patients with X-linked agammaglobulinaemia were predominantly male (83.3% vs. 16.7%), whereas those with immunoglobulin subclass deficiencies were more often female (79% vs. 21%). However, it must be noted that only six patients had X-linked agammaglobulinaemia. Three patients reported they had been diagnosed with a psychiatric disease but did not specify the disease. Age-specific differences were only visible for the aspect that female patients were significantly older than male patients (median age 46 vs. 37 years, *p* = 0.006).

**Table 1:** Baseline characteristics in 143 patients with IEI

Number of patients	143
**Age**	
Age in years, mean (SD±)	45.4 (15.2)
< 40	63 (44.1)
40–65	64 (44.8)
≥ 65	16 (11.2)
**Sex**	
Female, n (%)	88 (61.5)
**Disease**	
Common variable immunodeficiency n (%)	96 (67.1)
Antibody subclass deficiency n (%)	19 (13.3)
X-linked agammaglobulinaemia n (%)	6 (4.2)
Secondary antibody deficiency n (%)	5 (3.5)
Other n (%)	17 (11.9)
**Mental illness n (%)**	49 (34.3)^1^
Depression n (%)	31 (67.4)
Anxiety/Panic disorder n (%)	9 (19.5)
Other n (%)	6 (13.0)
**Sleep disorder n (%)**	58 (40.6)
**Social isolation n (%)**	79 (56)
Social isolation permanently n (%)	16 (11.4)
Social isolation occasionally n (%)	63 (45.0)
Social isolation never n (%)	61 (43.6)

Data are presented as mean ± standard deviation (SD) for continuous variables and as frequencies (n) and percentages (%) for categorial variables. Patients who did not fully complete the PHQ-9 or GAD-7 questionnaire were excluded from this analysis

^1^Among the 49 participants reporting a mental illness, 46 provided information on the specific diagnosis; the remaining 3 participants did not disclose their diagnosis

### Aspects of isolation

Of the 143 IEI patients, 140 responded to questions about self-isolation due to concerns about infection. Of these, 79 (56.4%) reported self-isolation due to concerns about infection. Sixteen (11.4%) patients isolated themselves permanently while 63 (45%) patients did so occasionally. Reported self-isolation was significantly associated with poor control of infection susceptibility (χ² = 8.8, *p* = 0.012) and pre-existing psychiatric comorbidity (χ² = 15.4, *p* < 0.001). Patient reported outcomes such as pain, quality of life and fatigue, each measured using VAS (Table 2, Figure S7-S9), were higher when patients isolated themselves (all *p* < 0.001). Post-hoc analysis also revealed significant differences between frequency subgroups. No significant association was revealed between isolation and sex (Fisher’s exact test, *p* = 0.362).

**Table 2:** Effects of self-isolation on different outcome variables

Median (IQR)	Isolation permanently	Isolation occasionally	Isolation never
PHQ-9	13.5 (7.75)	7 (7)	3 (5)
GAD-7	11 (8.5)	7 (7)	3 (5)
Sleep disturbance [1]	7 (4)	3.75 (5)	4.25 (5.5)
Pain intensity	4.5 (6.75)	3 (4)	0 (2)
QOL	6 (2)	4 (3)	3 (6)
Fatigue intensity	7 (3.75)	4 (5)	2 (5)

Data are presented as median (interquartile range, IQR) for continuous variables and as frequencies (n) and percentages (%) for categorial variables. PHQ-9 = Patient Health Questionnaire-9; GAD-7 = Generalized Anxiety Disorder-7; QOL = quality of life. Pain intensity, fatigue intensity and quality of life were assessed using a visual analogue scale (VAS) and sleep disturbance was assessed as a binary variable

### Susceptibility to infections

Of the 143 IEI patients, 140 answered questions about susceptibility to infection. 116 (82.9%) patients reported a subjectively increased susceptibility, while 24 (17.1%) reported no increased susceptibility. The mean number of infections in the preceding six months was 1.31 (IQR 2). At least four infections in the preceding six months were reported by 13 (9.1%) patients. Although the patients’ and physicians’ assessments of infection susceptibility control were consistent in 78.6% of cases, the overall inter-rater reliability was low (Cohen’s κ = 0.1, 95% CI − 0.09–0.28, *p* = 0.232). Furthermore, no significant association was seen between patients with preexisting psychiatric comorbidities and self-reported susceptibility control (OR 0.55, 95% CI 0.23–1.35, χ² = 1.71, *p* = 0.190). No significant sex-specific differences were found regarding susceptibility to infection (both subjective [OR 0.49, 95% CI 0.18–1.32, χ² = 2.04, *p* = 0.154] and measured by the number of infections in six months [Figure S1, *p* = 0.337]). Of notice, all other patient-reported outcomes (pain, quality of life, fatigue and PHQ-9 and GAD-7 scores) were significantly associated with the control of infection susceptibility (Figure S2-S6).

### Frequency and severity of depressive and anxiety symptoms

All patients were asked to fill the PHQ-9 and GAD-7 questionnaire, and 143 IEI patients provided information regarding their depressive and anxiety symptoms. The median pain score (VAS) was 2 (IQR 5), the mean quality of life score (VAS) was 4 (IQR 5). The median PHQ-9 score was 5 (IQR 8) and the GAD-7 was 5 (IQR 7).

Eighty-one (56.6%) patients achieved a minimum of 5 points on the PHQ-9 questionnaire, which corresponds to at least mild depressive symptoms. A score of at least 10 was achieved by 40 (28%) patients, indicating at least moderate symptoms. Eighteen (12.6%) patients exceeded 15 points, indicating at least moderately severe symptoms, while 5 (3.5%) patients achieved at least 20 points, and therefore showed severe depressive symptoms.

Of the patients assessed for anxiety symptoms using the GAD-7 questionnaire, 75 (52.5%) patients had at least mild symptoms (scores ≥ 5), 33 (23.1%) had at least moderate symptoms (scores ≥ 10), and 16 (11.2%) had severe anxiety (scores ≥ 15). Table 3 provides an overview of PHQ-9 and GAD-7 scores.

**Table 3:** Grouped PHQ-9 and GAD-7 scores as well as pain, QOL and fatigue

	All Patients	Patients with known psychiatric comorbidity
	*n* = 143	*n* = 49
**PHQ-9 Score completed**		
0–4 none n (%)	62 (43.4)	6 (12.2)
5–9 mild n (%)	41 (28.7)	14 (28.6)
10–14 moderate n (%)	22 (15.4)	15 (30.6)
15–19 moderately severe n (%)	13 (9.1)	9 (18.4)
20–27 severe n (%)	5 (3.5)	5 (10.2)
≥ 10 Points n (%)	40 (28)	29 (59.2)
**GAD-7 Score completed**	*n* = 143	*n* = 49
0–4 none n (%)	68 (47.6)	8 (16.3)
5–9 mild n (%)	42 (29.4)	16 (32.7)
10–14 moderate n (%)	17 (11.9)	13 (26.5)
15–21 severe n (%)	16 (11.2)	12 (24.2)
≥ 10 Points n (%)	33 (23.1)	25 (51)
**Pain (VAS)** median (IQR)	2 (5)	5 (5)
**QOL (VAS)** median (IQR)	4 (4)	5 (3)
**Fatigue (VAS)** median (IQR)	4 (5.3)	7 (3.3)

Data are presented as median (interquartile range, IQR) for continuous variables and as frequencies (n) and percentages (%) for categorial variables. PHQ-9 = Patient Health Questionnaire-9; GAD-7 = Generalized Anxiety Disorder-7; VAS = visual analogue scale; QOL = quality of life. Clinically relevant depressive and anxiety symptoms were defined as PHQ-9 ≥ 10 and GAD-7 ≥ 10, respectively. The column “patients with known psychiatric comorbidity” refers to participants with a previously diagnosed psychiatric disorder

As expected, PHQ-9 and GAD-7 scores were significantly higher in patients with either a known psychiatric disease (Figure S10, S11) or with sleep disturbances (Figure S12, S13), as determined by the Wilcoxon–Mann–Whitney test (both *p* < 0.001). In contrast, the current treatment of mental illness had no significant effect on PHQ-9 (*p* = 0.992) and GAD-7 scores (*p* = 0.269).

A considerable proportion of patients with moderate to severe symptoms of anxiety or depression (defined as PHQ-9 or GAD-7 scores ≥ 10, respectively) had no documented psychiatric diagnosis and therefore received no treatment. Eleven out of 40 patients (27.5% [95% CI 0.16–0.43]) scored 10 or more points on the PHQ-9 questionnaire but did not report a known psychiatric disorder. Similarly, eight out of 33 patients (24.2% [95% CI 0.13–0.41]) scored 10 or more points on the GAD-7 questionnaires but did not report a known psychiatric disorder. Overall, 26% of patients with a clinically relevant mental health burden received neither diagnosis nor treatment.

**Fig. 1:**
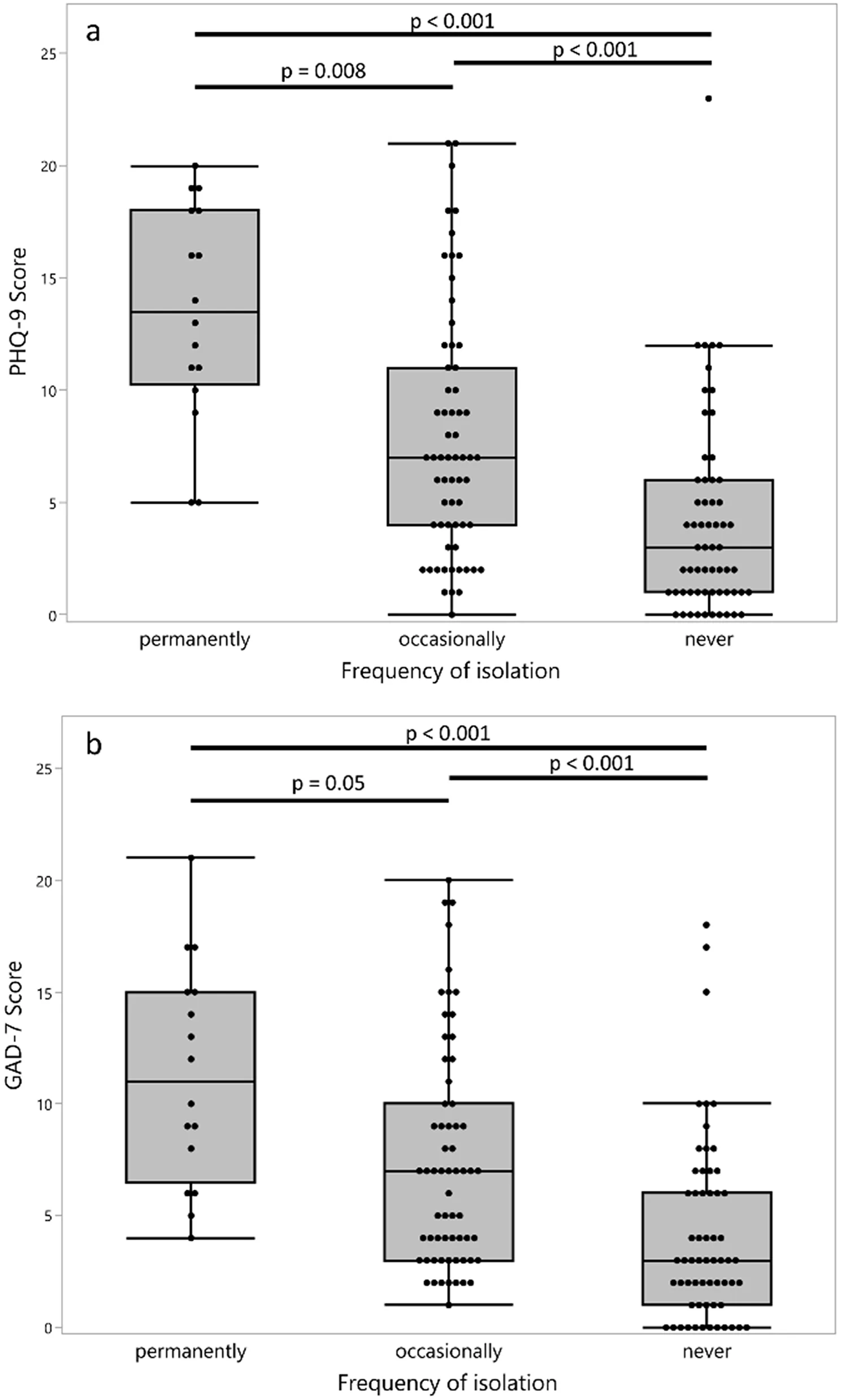
PHQ-9 and GAD-7 scores according to frequency of social Isolation. PHQ-9 (**a**) and GAD-7 (**b**) scores according to frequency of social isolation. boxplots display median, interquartile range, whiskers extend to the most extreme values within 1.5 × IQR. individual observations are displayed as jittered points. higher levels of isolation were associated with increased depressive symptom severity. group differences were assessed using the kruskal–wallis test followed by dunn–bonferroni post hoc correction. statistically significant differences were observed between groups (exact p-values)

Patients who isolate themselves due to concerns about infection had significantly higher PHQ-9 (Spearman’s ρ = 0.52, *p* < 0.001) and GAD-7 (Spearman’s ρ = 0.48, *p* < 0.001) scores (Fig. 1). As a secondary analysis, group comparisons revealed significant differences between patients who isolate themselves and those who do not (*p* < 0.001). Dunn’s post-hoc analysis revealed significant differences between all categories of isolation, particularly between those who permanently isolate and those who never isolate (both *p* < 0.001).

When subjective control of infection, based on patient-reported outcomes, is compared with the objective assessment by the treating physicians, subjective infection control is associated with higher PHQ-9 (*p* < 0.001 vs. 0.33) and GAD-7 (*p* = 0.007 vs. 0.18) scores (Figure S14, S15). However, the objective assessment is not associated with increased mental distress.

No significant sex-specific differences were found regarding the PHQ-9 and GAD-7 scores neither for pain, nor quality of life or fatigue.

**Fig. 2:**
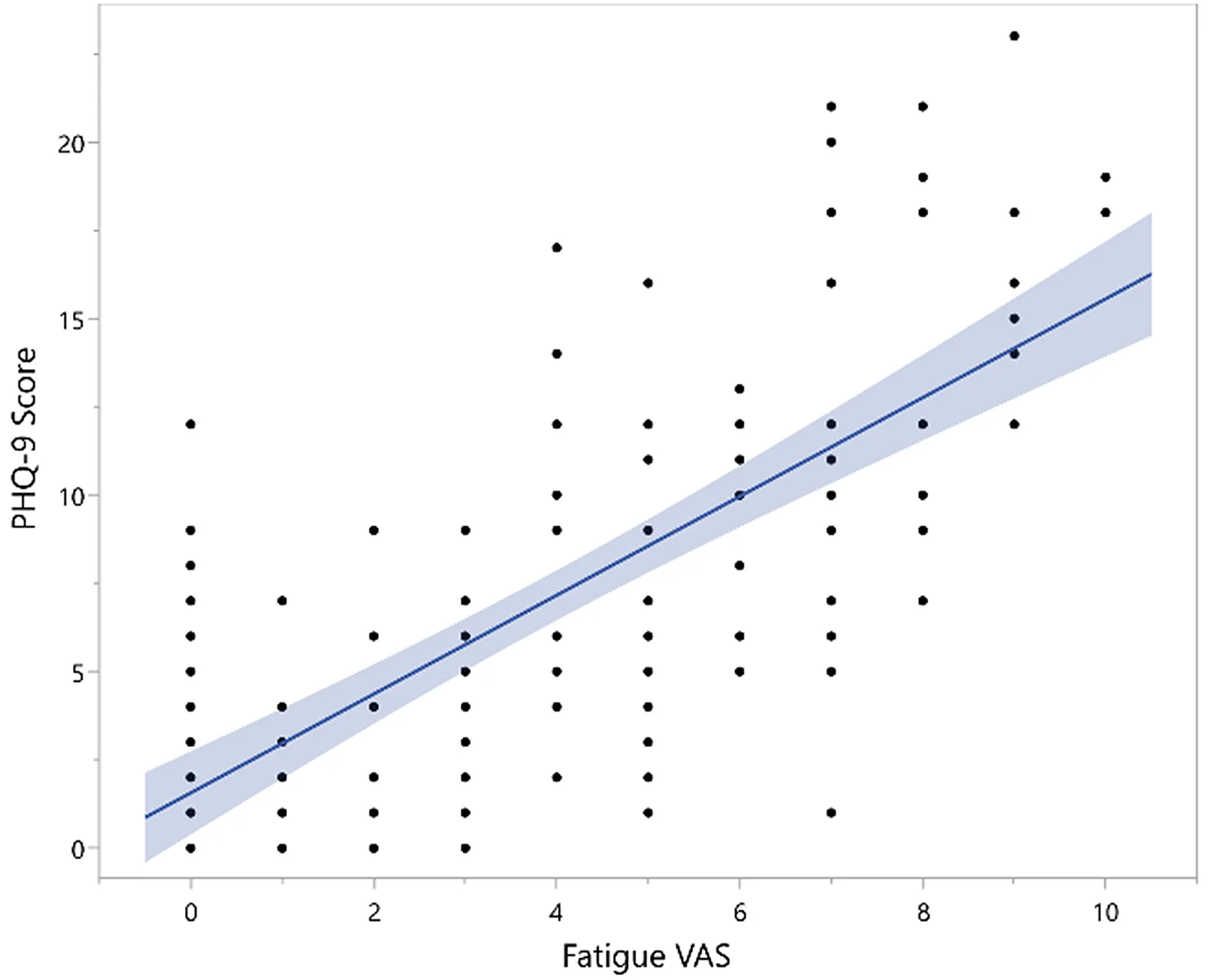
Association between fatigue severity (VAS) and depressive symptoms (PHQ-9). Scatter plot with linear regression line and 95% confidence interval. higher fatigue levels were associated with increased PHQ-9 scores

To assess independent associations between clinical and psychosocial factors and depressive and anxiety symptom severity, linear regression analyses were conducted with PHQ-9 and GAD-7 scores as outcome variables. Higher pain intensity was significantly associated with greater depressive symptom burden, measured with PHQ-9 scores (b = 1.18, 95% CI 0.89–1.46, *p* < 0.001), explaining 32% of the variance (R² = 0.32, F (1,140) = 66.63, *p* < 0.001). PHQ-9 scores were also significantly associated with fatigue intensity (b = 1.40, 95% CI 1.16–1.64, *p* < 0.001) and quality of life (b = 0.98, 95% CI 0.61–1.34, *p* < 0.001) in separate linear regression models. Fatigue (Fig. 2) explained 50.7% of the variance in PHQ-9 scores (R² = 0.51, F (1,128) = 131.8, *p* < 0.001), whereas quality of life only explained 17% (R² = 0.17, F (1,139) = 28.52, *p* < 0.001). However, significant lack of fit was observed for both models (fatigue: *p* < 0.001; quality of life: *p* = 0.032), indicating that linear models may not fully capture these associations.

**Fig. 3:**
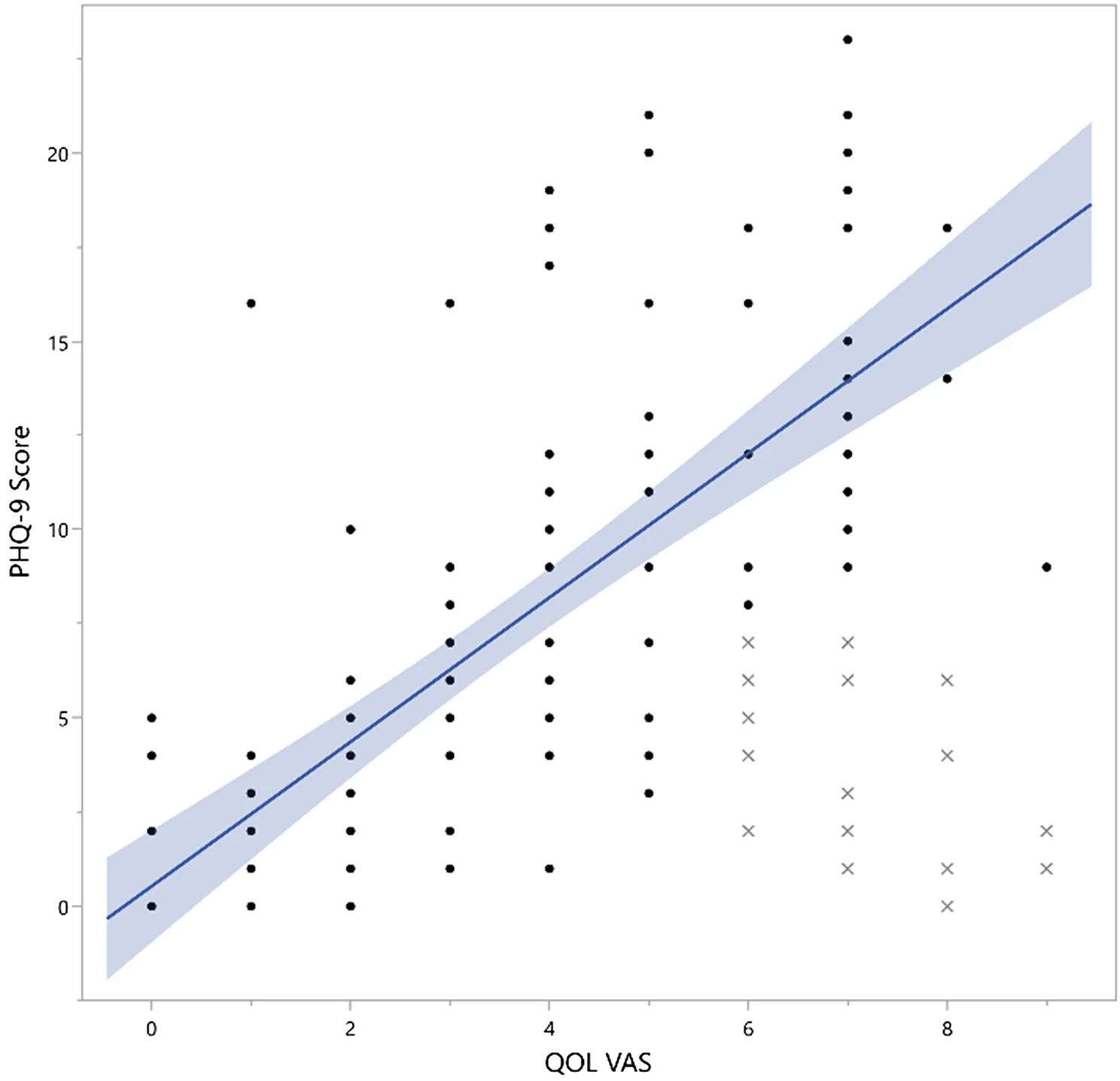
Sensitivity analysis: association between quality of life (VAS) and depressive symptoms (PHQ-9). sensitivity analysis: scatter plot with linear regression line and 95% confidence interval. worse quality of life was associated with increased PHQ-9 scores. grey crosses indicate excluded observations

Inspection of the scatter plot suggested that a subset of patients may have interpreted the QOL VAS in the opposite direction, as indicated by responses showing unexpectedly low PHQ-9 scores at higher QOL VAS values. These participants were marked with crosses in Fig. 3 and excluded in exploratory sensitivity analysis. In the restricted sample, the linear regression model no longer showed evidence of lack of fit (Fig. 3). Higher QOL VAS scores were associated with higher PHQ-9 scores (b = 1.92, 95% CI 1.56–2.27, *p* < 0.001; R² = 0.50, F (1,117) = 116.56, *p* < 0.001).

When modelling GAD-7 scores using linear regression, significant lack of fit was observed for several models indicating deviations from linearity. Therefore, associations were assessed using Spearman’s rank correlation coefficients. Higher fatigue (ρ = 0.62, *p* < 0.001), greater pain intensity (ρ = 0.53, *p* < 0.001), and poorer quality of life (ρ = 0.41, *p* < 0.001) were significantly associated with higher GAD-7 scores.

**Fig. 4:**
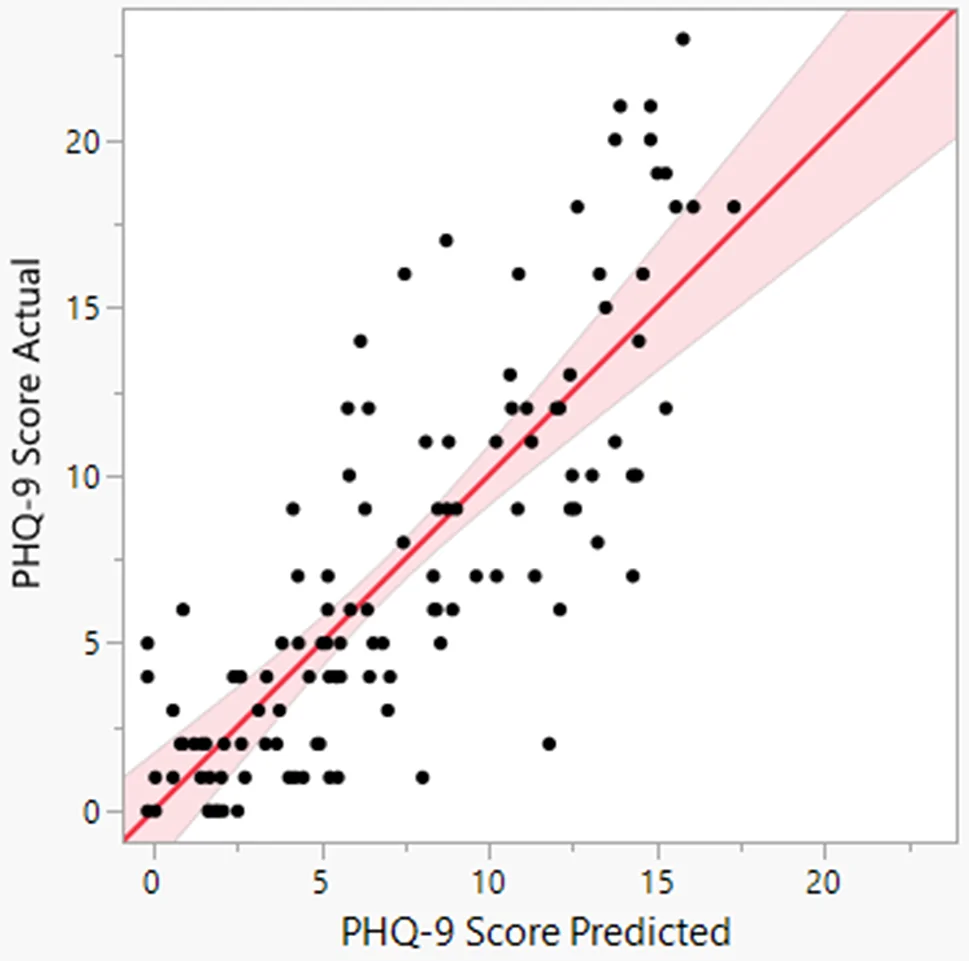
Actual vs. predicted PHQ-9 scores (full multivariable model). actual versus predicted PHQ-9 scores based on the full multivariable linear regression model. pain, fatigue, quality of life, diagnosed psychiatric disorder, and sleep disturbances were included as independent predictors (R² = 0.68, *p* < 0.001). the red line (with 95% confidence interval) represents the identity line, representing perfect agreement between predicted and observed PHQ-9 scores

Using multivariable linear regression analyses, pain, fatigue, quality of life, diagnosed psychiatric comorbidity as well as sleep disturbances and self-isolation were identified as independent predictors of higher PHQ-9 scores (Fig. 4; Table 4), with the model explaining 67.9% of the variance (R² = 0.68, F(6,120) = 42.42, *p* < 0.001). An exploratory backward stepwise regression was performed, starting with the full prespecified model. Model reduction was guided by the minimum Bayesian information criterion (BIC) to identify a sparser model. The predictors quality of life and self-isolation were therefore excluded (R² = 0.65, F(4,125) = 58.67, *p* < 0.001). Both models showed no significant lack of fit (*p* = 0.11 and *p* = 0.175). The Durbin–Watson statistics were 2.07 and 2.17, respectively, indicating no evidence of relevant first-order autocorrelation in the predictors. Variance Inflation Factors were consistently < 5, indicating no relevant multicollinearity.

**Table 4:** Multivariable linear regression analysis for PHQ-9

Predictor	Full model: b (95% CI), *p*, VIF	Backward-BIC model: b (95% CI), *p*, VIF
Fatigue intensity	0.75 (0.47–1.03), *p* < 0.001, 1.94	0.81 (0.55–1.09), *p* < 0.001, 1.74
Sleep disturbance [0]	–1.48 (–2.23 to − 0.72), *p* < 0.001, 1.56	–1.56 (–2.29 to − 0.84), *p* < 0.001, 1.41
Psychiatric comorbidity [0]	–1.12 (–1.84 to − 0.41), *p* < 0.001, 1.35	–1.10 (–1.83 to − 0.38), *p* = 0.003, 1.34
Pain intensity	0.29 (0.02–0.58), *p* = 0.04, 1.67	0.35 (0.07 to − 0.63), *p* = 0.014, 1.63
Quality of life	0.23 (–0.04 to − 0.50), *p* = 0.093, 1.24	Not retained
Isolation [0]	–0.42 (–1.11 to 0.28), *p* = 0.236, 1.33	Not retained

b represents unstandardized regression coefficient. CI = confidence interval. Higher scores of fatigue and pain intensity indicate greater symptom severity, whereas higher scores of quality of life indicate worse overall well-being. Psychiatric comorbidity, sleep disturbance and isolation were coded as a binary variable

**Fig. 5:**
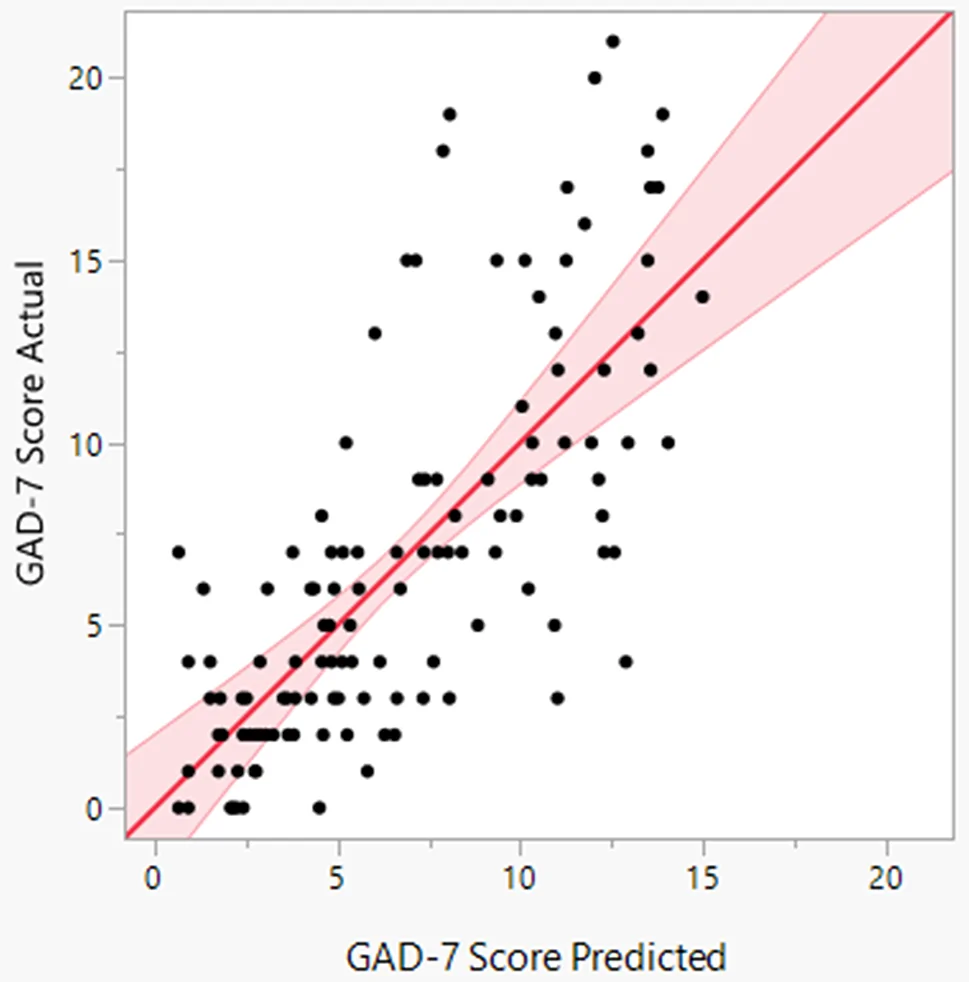
Actual vs. predicted GAD-7 scores (full multivariable model). actual versus predicted GAD-7 scores based on the multivariable linear regression model. pain and fatigue intensity, psychiatric comorbidity and sleep disturbances were included as independent predictors (R² = 0.52, *p* < 0.001). The red line (with 95% confidence interval) represents the identity line, representing perfect agreement between predicted and observed GAD-7 scores

As illustrated in Fig. 5 and detailed in Table 5, pain and fatigue intensity, psychiatric comorbidity, and sleep disturbances were identified as independent predictors of GAD-7 scores, with the model explaining 57% of the variance (R² = 0.57, F(6,120) = 26.39, *p* < 0.001). As for PHQ-9, an exploratory backward stepwise regression was performed. The predictors quality of life and self-isolation were therefore not retained (R² = 0.52, F(4,125) = 34.47, *p* < 0.001). Both models showed no significant lack of fit (*p* = 0.06 vs. *p* = 0.07). The Durbin–Watson statistics were 2.1 and 1.96, respectively, indicating no evidence of relevant first-order autocorrelation in the predictors. Variance Inflation Factors were consistently < 5, indicating no relevant multicollinearity.

**Table 5:** Multivariable linear regression analysis for GAD-7

Predictor	Full model: b (95% CI), *p*, VIF	Backward-BIC model: b (95% CI), *p*, VIF
Pain intensity	0.33 (0.04–0.62), *p* = 0.026, 1.67	0.39 (0.1–0.69), *p* = 0.009, 1.63
Fatigue intensity	0.41 (0.12–0.71), *p* = 0.006, 1.94	0.49 (0.2–0.77), *p* = 0.001, 1.74
Psychiatric comorbidity [0]	−1.19 (–1.94 to − 0.44), *p* = 0.002, 1.35	−1.18 (–1.95 to − 0.41), *p* = 0.003, 1.34
Sleep disturbance [0]	–1.25 (–2.05 to − 0.47), *p* = 0.002, 1.56	−1.3 (–2.07 to − 0.53), *p* = 0.001, 1.41
Quality of life	0.27 (–0.01–0.55), *p* = 0.066, 1.24	Not retained
Isolation [0]	–0.44 (–1.16–0.29), *p* = 0.236, 1.33	Not retained

b represents unstandardized regression coefficient. CI = confidence interval. Higher scores of fatigue and pain intensity indicate greater symptom severity, whereas higher scores of quality of life indicate worse overall well-being. Psychiatric comorbidity, sleep disturbance and isolation were coded as a binary variable

## Discussion

Mental distress is common and clinically relevant in patients with IEI. According to previous studies, depressive symptoms appear to be more prevalent among individuals experiencing social isolation compared with those who do not self-isolate [27]. In line with this, our findings indicate that a considerable proportion of patients with IEI report socially isolating themselves due to concerns about infections. At the same time, screening with validated instruments for depressive and anxiety symptoms (PHQ-9 and GAD-7) revealed a substantial symptom burden in this cohort, exceeding the reported prevalence in the general German population [13]. Notably, despite this elevated level of depressive and anxiety symptoms, a considerable proportion of patients did not report a formally diagnosed psychiatric disorder, suggesting potential under-recognition or undersupply of mental health care in this population. The implementation of standardized screening instruments, similar to those routinely used in other chronic diseases at the time of diagnosis and during follow-up, may facilitate earlier identification and management of mental health comorbidities [28].

Although acute COVID-19-related isolation measures had ended by the time of data collection (July 2024 to February 2025), the pandemic may have had persistent effects on infection-avoidance behaviours due to infection concerns particularly in individuals vulnerable to infectious diseases [28, 29]. As we did not assess pre-pandemic of longitudinal isolation behaviour, we cannot determine to what extent the observed self-isolation reflects persistent pandemic-related behavioural changes, IEI-specific infection concerns or other individual factors. Accordingly, the observed association between self-isolation and psychological symptom burden should not be interpreted as IEI-specific [29, 30].

The actual risk of infection was not objectively assessed in this study; therefore, the patients’ subjective perception of risk was taken into account. Patients may isolate themselves due to excessive anxiety even without a thread of infection representing a potential confounding factor.

Depressive symptoms, as measured using the PHQ-9, were associated with fatigue severity. This association should be interpreted with caution because the PHQ-9 includes an item on fatigue and low energy, resulting in a partial symptom overlap. In addition, fatigue may be independently associated with IEI, such that a greater physical illness burden could contribute to elevated PHQ-9 scores without necessarily indicating a distinct depressive disorder. Furthermore, systemic use of corticosteroids can affect psychiatric symptom levels [31] but were not used in our cohort as a routine component of IEI management.

Some general limitations of this study should be considered when interpreting the findings. We used established self-rating questionnaires for anxiety and depression and can therefore only comment on the extent of subjective symptom intensity which does not involve a clinical diagnosis. Due to the cross-sectional study design, causal inferences cannot be drawn, or only to a very limited extent. Furthermore, the range of assessed sociodemographic variables such as income, educational and employment status, and living situation, was limited, which may restrict the generalisability of the results. Additionally, numerous factors that may influence mental health, such as religiosity, social support networks, and spirituality were not assessed. Therefore, the lack of a matched control group should be acknowledged when comparing levels of mental distress in our population with those in the general population. Although selection bias cannot be excluded, referral to our centre may represent limited regional access to facilities with expertise in managing IEI, rather than greater symptom severity or anxiety alone. Our cohort was recruited from a single centre and was predominantly composed of patients with CVID (67.1%) so this limits generalisability to other IEI subtypes with different clinical profiles.

The low agreement between patient’s perceived infection control and physicians’ assessments (Cohen’s κ = 0.1, *p* = 0.232) suggests that subjective perceptions of infection susceptibility may differ substantially from the clinically assessed risk. Anxiety and depressive symptoms may contribute to an amplified perception of the risk of infection. Previous studies have shown that the interpretation of the patients’ own health status and disease severity may diverge from physician-reported outcomes in CVID patients [32]. One possible explanation is that common, uncomplicated infections were not included in the physicians’ assessment, although patients may consider such infections as evidence of increased susceptibility. This highlights the importance of addressing both objectively assessed infection risk as well as patients’ subjective concerns during clinical consultations. However, due to the cross-sectional design of this study, no conclusions can be drawn about the direction of the association.

Although IEI represents a heterogenous group of disorders with varying disease activity, susceptibility to infection, and psychosocial impact, the limited sample size did not allow for stratified analysis by disease subtype or disease severity.

The primary regression analysis suggested that the association between QOL VAS and depressive symptoms, as measured with PHQ-9, was not adequately described by a simple linear model in the full sample. Although a cubic model provided a better statistical fit, the nonlinearity was largely driven by a small subgroup of responses that may reflect heterogenous interpretation of the QOL VAS direction. After excluding these potentially misinterpreted responses, the linear model no longer showed evidence of lack of fit and a positive association between QOL VAS and PHQ-9 scores. However, because the exclusion was based on an exploratory assessment of response patterns, this result should be interpreted as a sensitivity analysis rather than evidence that the excluded responses were incorrect. These findings also highlight the importance of clear definition of visual analogue scales in future studies.

Given the reliance on self-reported measures, reporting bias cannot be ruled out, particularly regarding sensitive and potentially stigmatized topics such as mental health and infectious diseases. Despite these limitations, this study provides important real-world data on the mental burden of patients with IEI and highlights the need for better awareness for mental health in those patients.

Previous studies on the psychosocial burden of IEI have mainly focused on impaired quality of life, depression, and fatigue, whereas social isolation has received less attention. In our German cohort, permanent self-isolation was associated with increased symptom burden of depression and anxiety. These findings add a country-specific perspective to the existing literature and suggest that social isolation may represent an important factor regarding mental health in patients with IEI.

Physicians may encourage patients to balance the potential benefits of reduced social contact with the risks of prolonged social isolation. Current guidelines predominantly focus on medical strategies to reduce infection risk, whereas explicit recommendations addressing psychosocial support remain limited.

## Conclusions

Mental distress is common and clinically relevant in patients with inborn errors of immunity, with a substantial proportion experiencing depressive and anxiety symptoms exceeding those observed in the general German population. Despite this, many affected individuals remain without a formal psychiatric diagnosis, indicating potential under-recognition and unmet mental health needs. The implementation of standardized screening for depressive and anxiety symptoms may support the identification of patients being at risk for mental health comorbidities with the aim of facilitating earlier identification and potentially improving the management of mental health comorbidities in patients with IEI. In this population, social isolation was associated with a higher prevalence of mental health comorbidities. Counselling on social isolation and patients’ individual risk of infection should be integrated into routine clinical practice.

## Supplementary Information

Below is the link to the electronic supplementary material.

Supplementary Material 1

Supplementary Material 2

## Data Availability

The datasets generated during and/or analysed during the current study are not publicly available due to privacy concerns related to the small, single-center cohort and the rare disease context, which may compromise participant confidentiality. Data are available from the corresponding author on reasonable request and with approval from the local ethics committee.

## Abbreviations

CVID: Common Variable Immunodeficiency
GAD-7: Generalized Anxiety Disorder-7
IEI: Inborn Errors of Immunity
IgG: Immunoglobulin G
IQR: Interquartile Range
OR: Odds Ratio
PHQ-9: Patient Health Questionnaire-9
QOL: Quality of Life
SD: Standard Deviation
VAS: Visual Analogue Scale
