# Supplementary material for: Frequency of depression and anxiety in patients with inborn errors of immunity: a cross-sectional study
Source: BMC Psychiatry. 2026 Sep 15;26:721. doi: 10.1186/s12888-026-08654-1 (PMC13576266; doi:10.1186/s12888-026-08654-1)
Supplement: Supplementary file 1 [file 12888_2026_8654_MOESM2_ESM.pdf]

Supplements

Legend

Box-and-whisker plots with overlaid individual data points are shown. The boxes represent the interquartile range (IQR) with the median indicated by the central line; whiskers extend to the most extreme values within 1.5 × IQR. Individual observations are displayed as jittered points.

For binary variables on the x-axis, values are coded as 0 = no and 1 = yes.

Figure S1

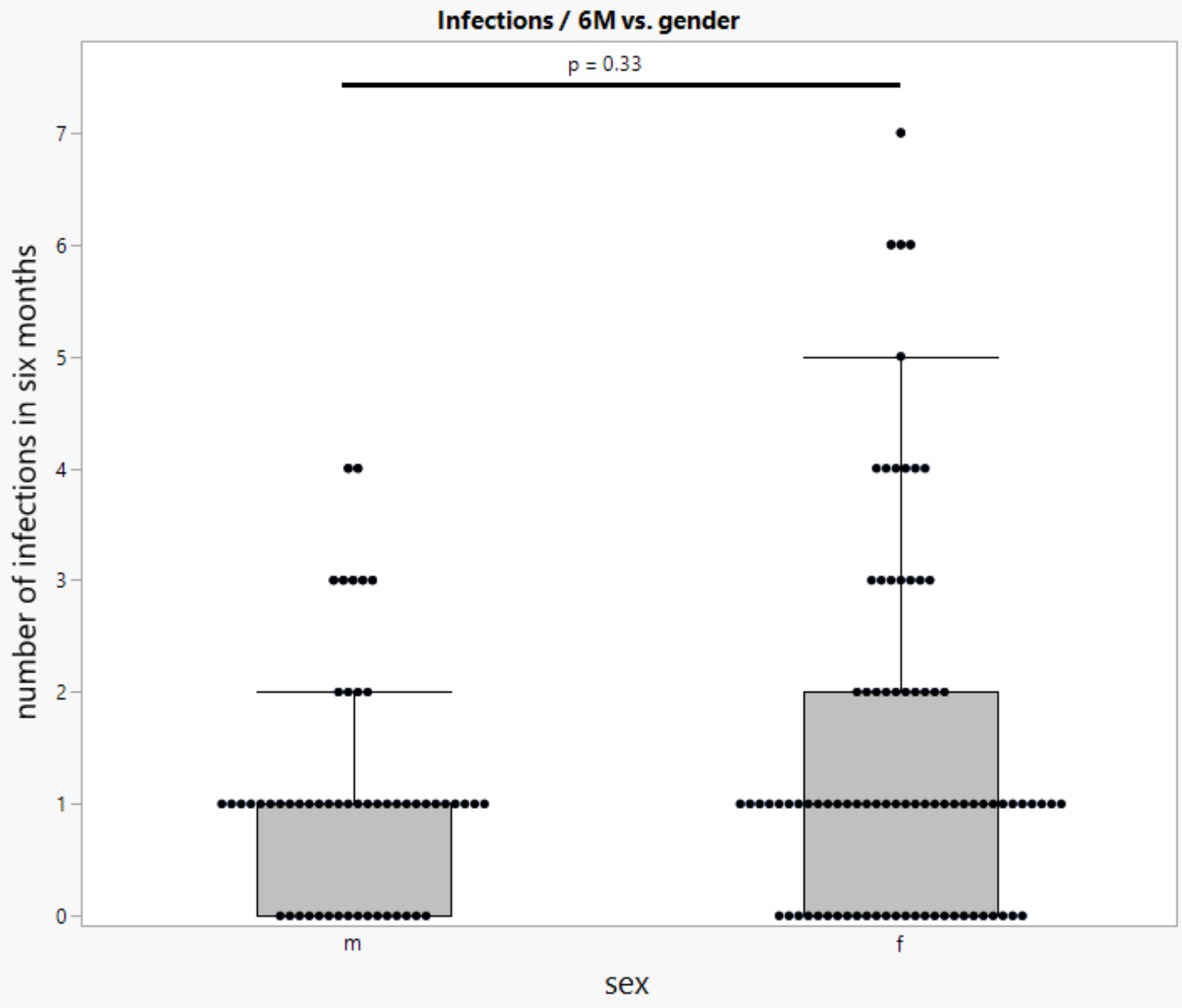

Figure S2

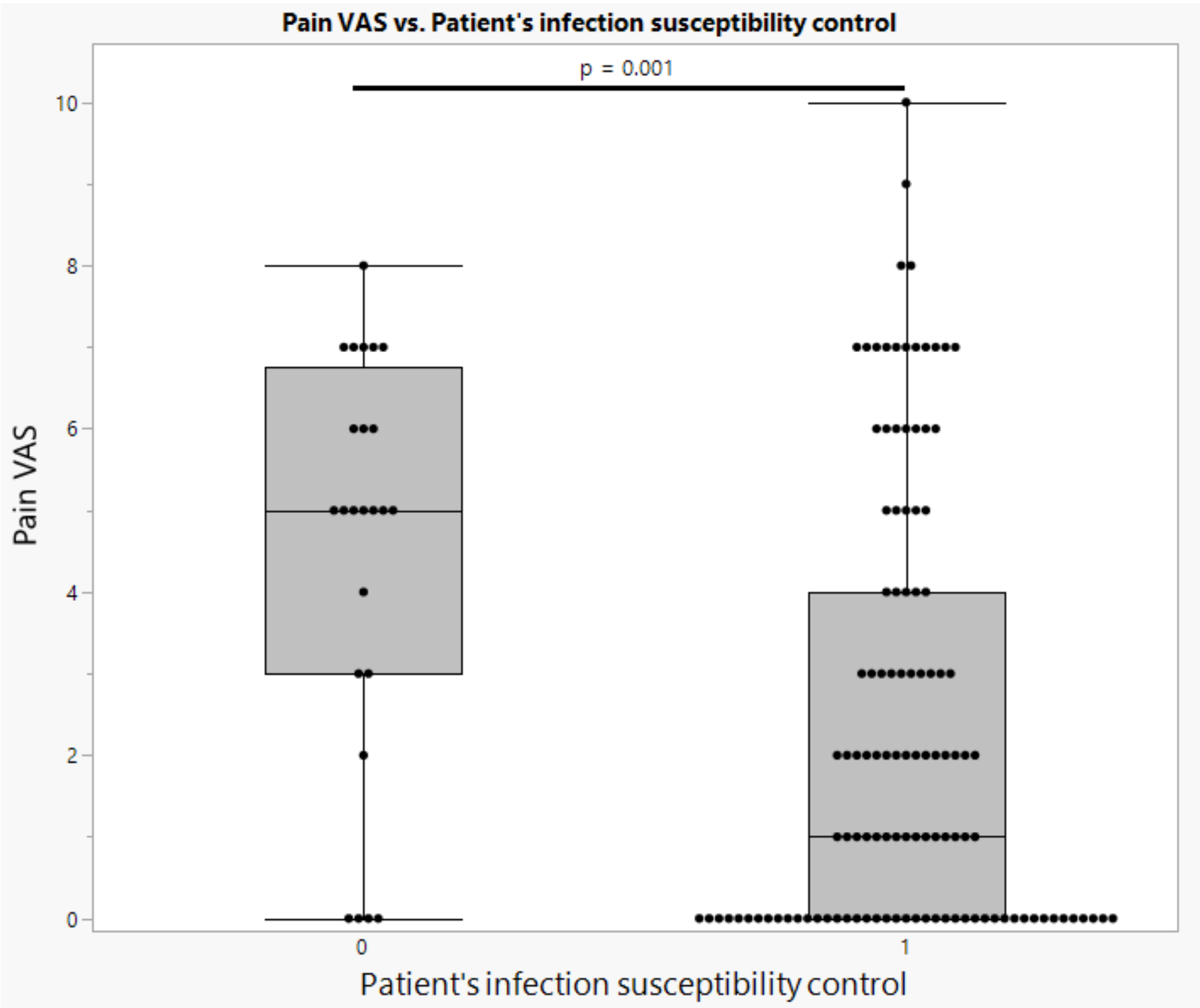

Figure S3

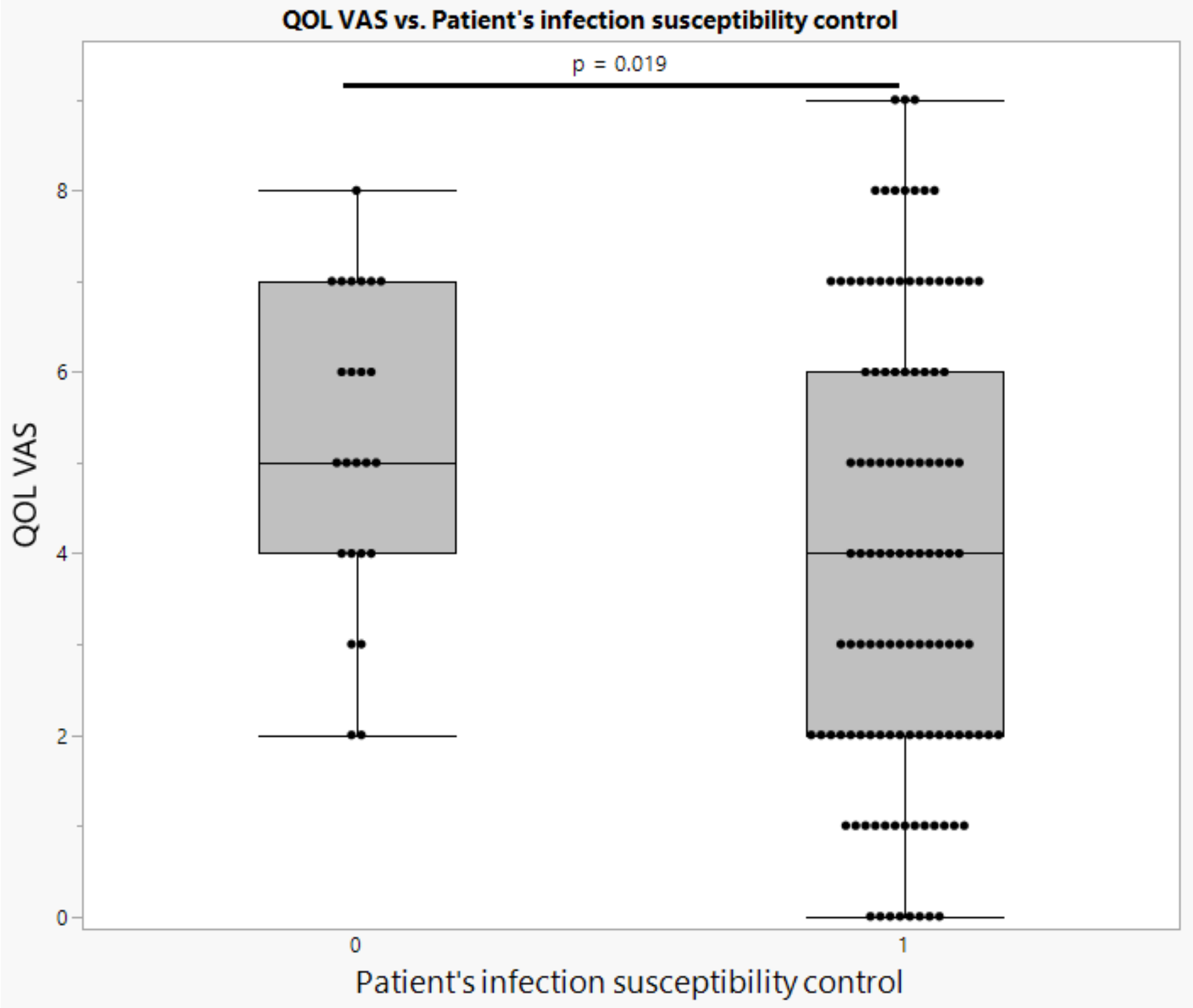

Figure S4

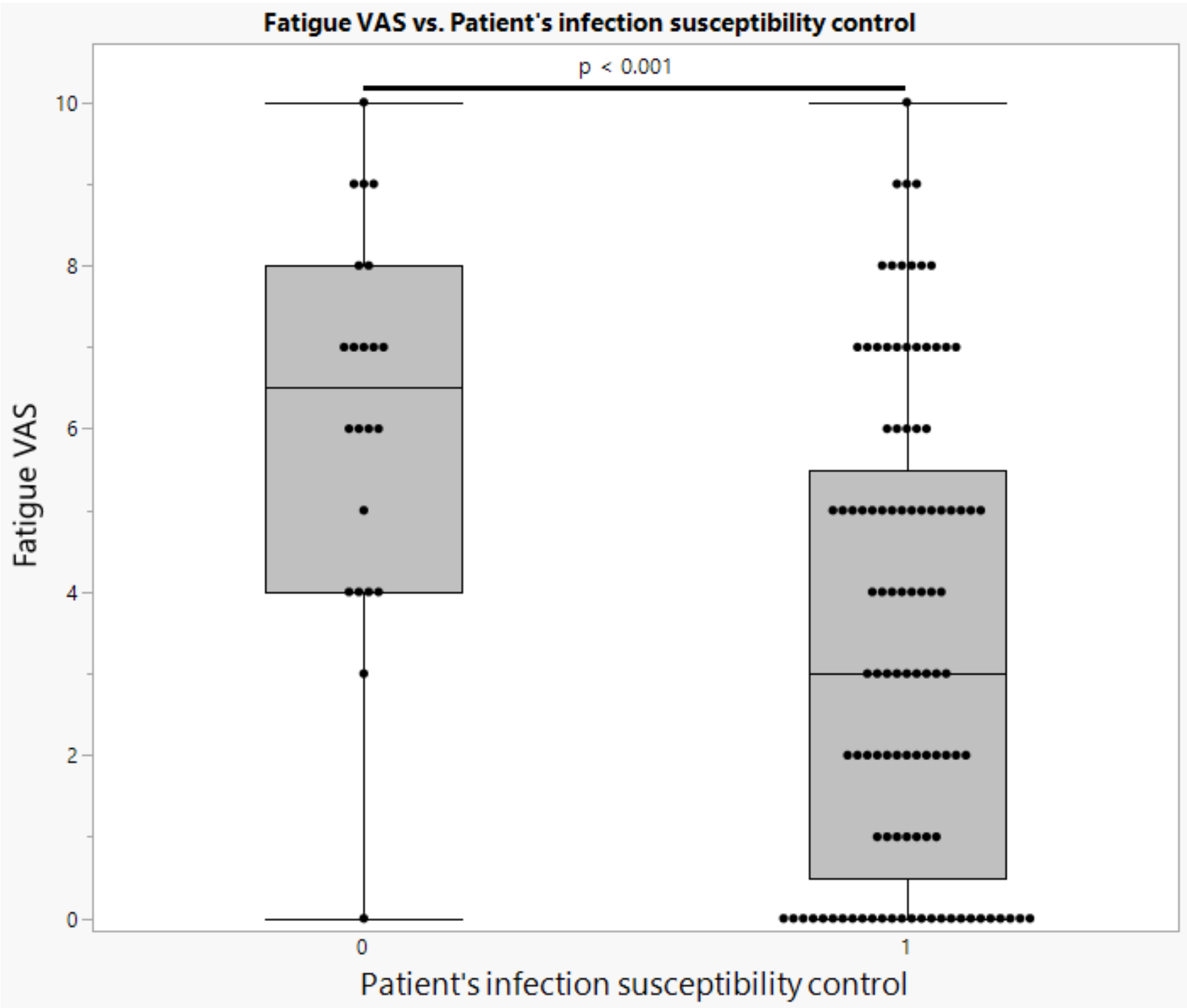

Figure S5

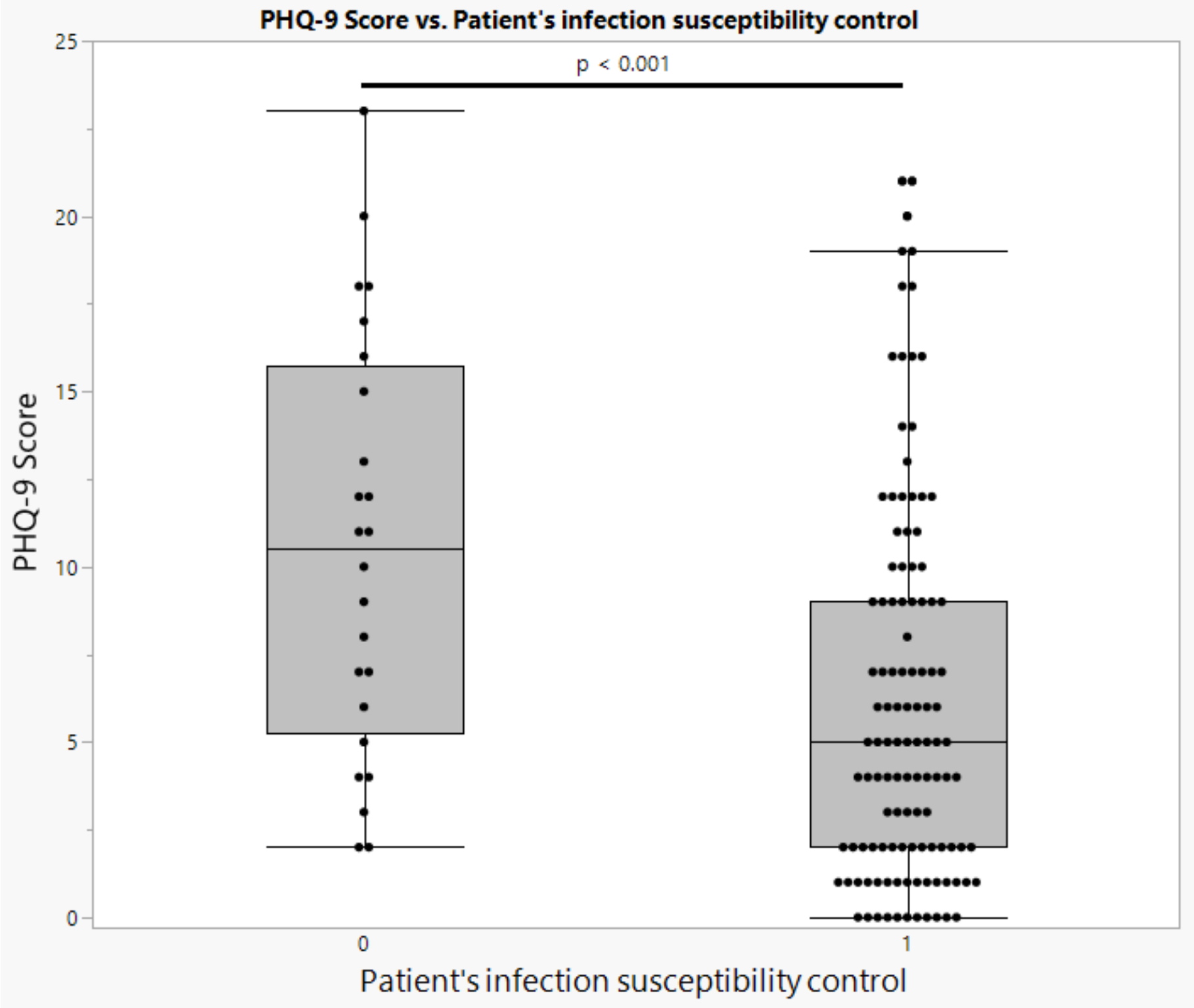

Figure S6

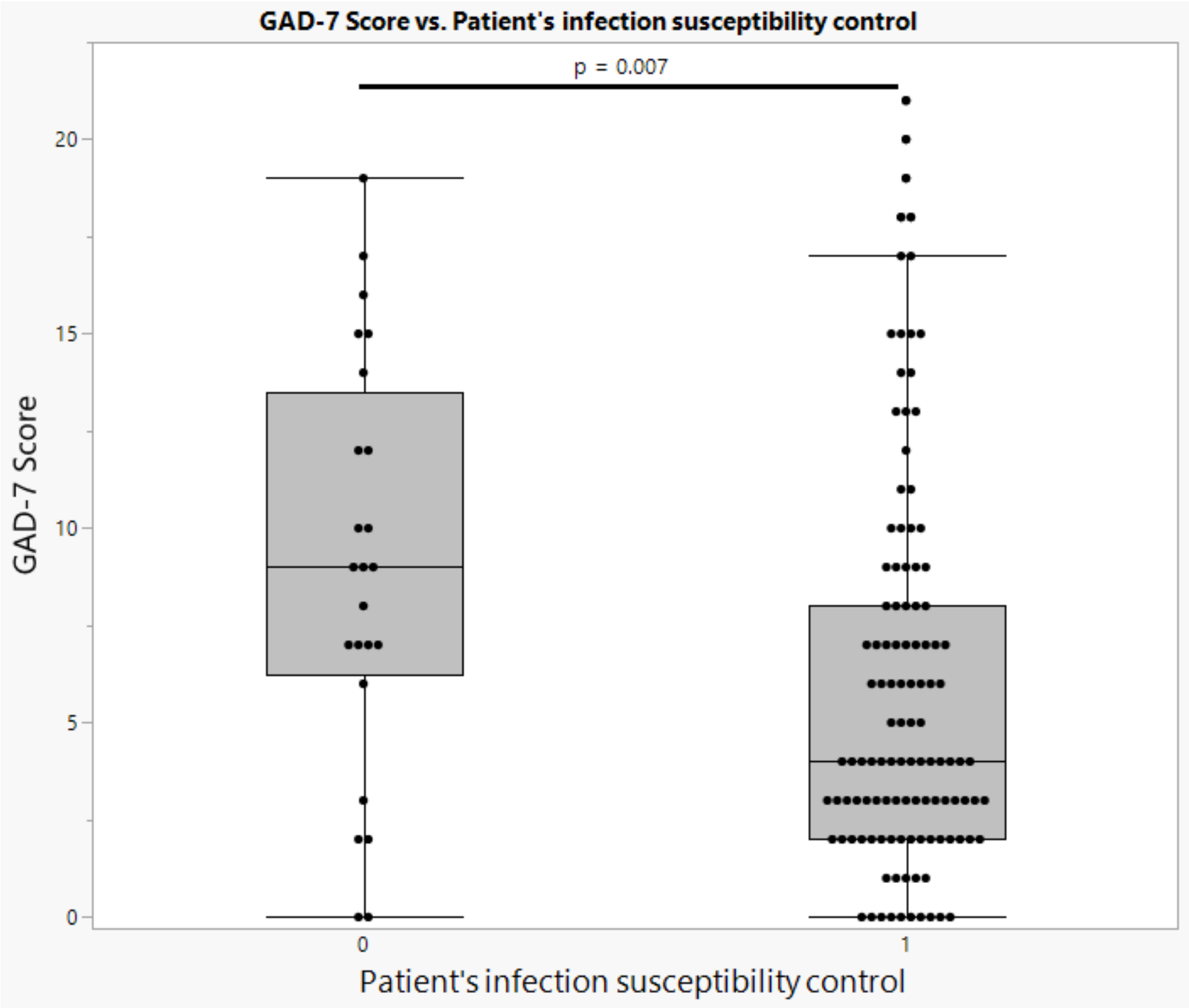

Figure S7

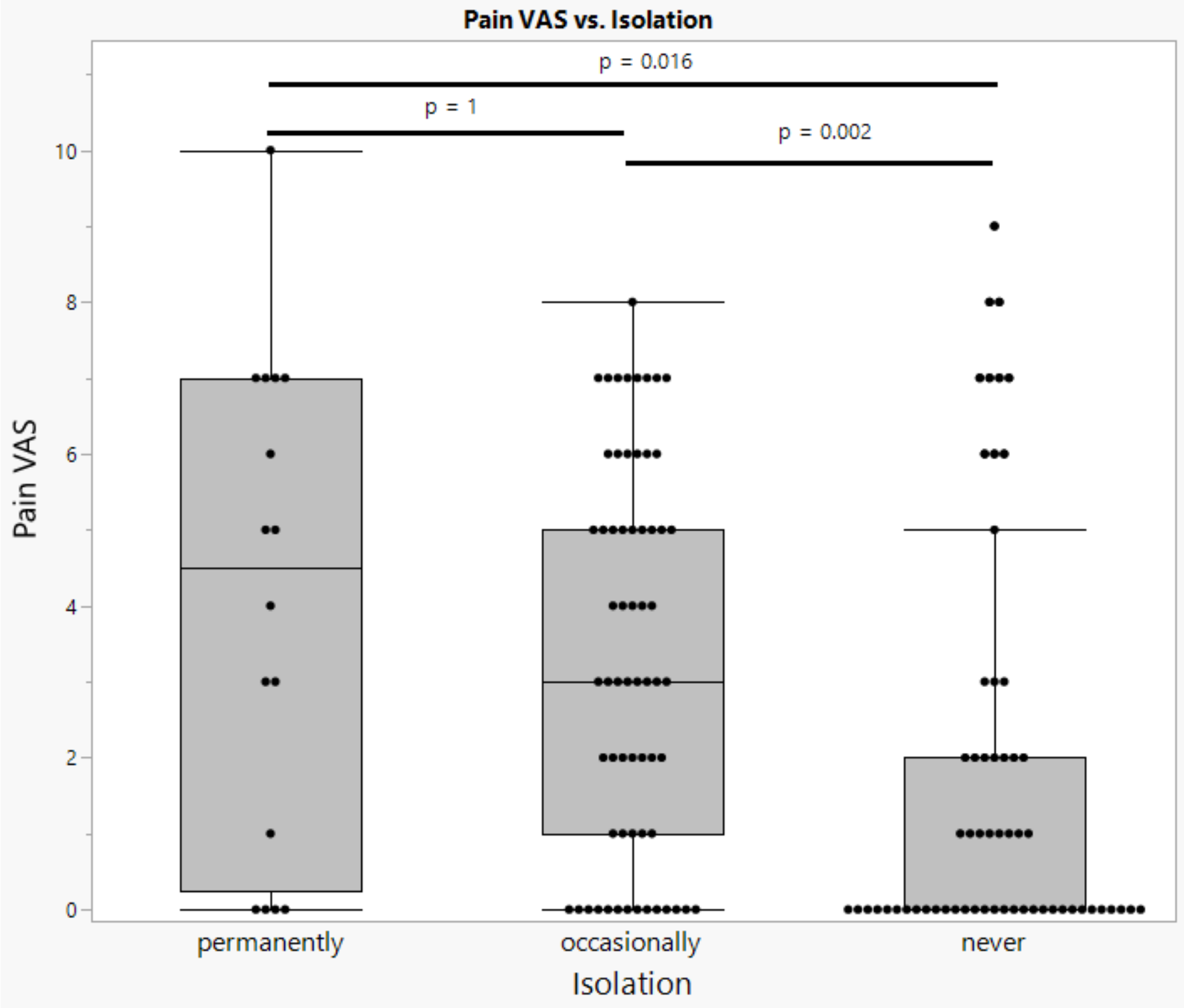

Figure S8

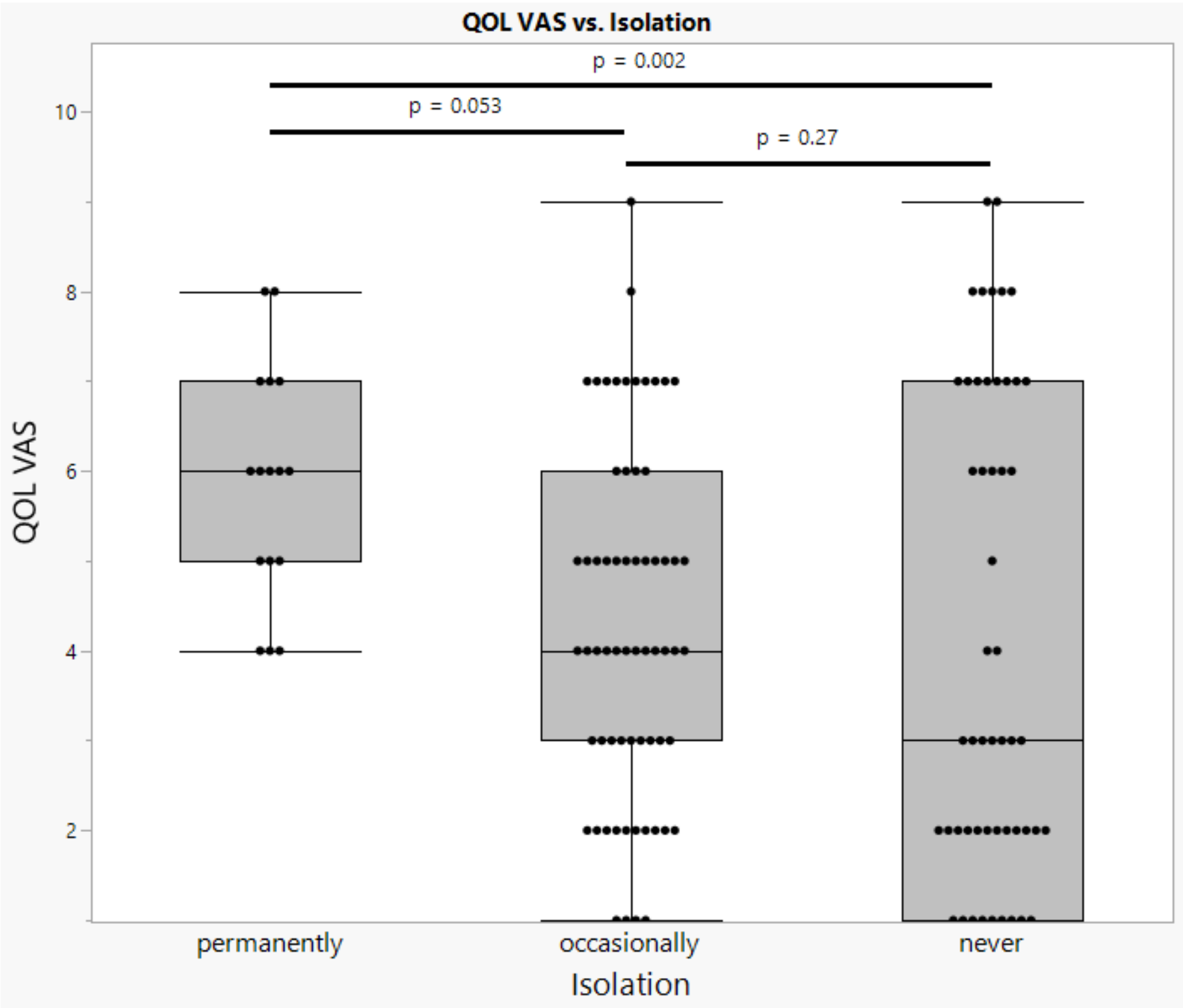

Figure S9

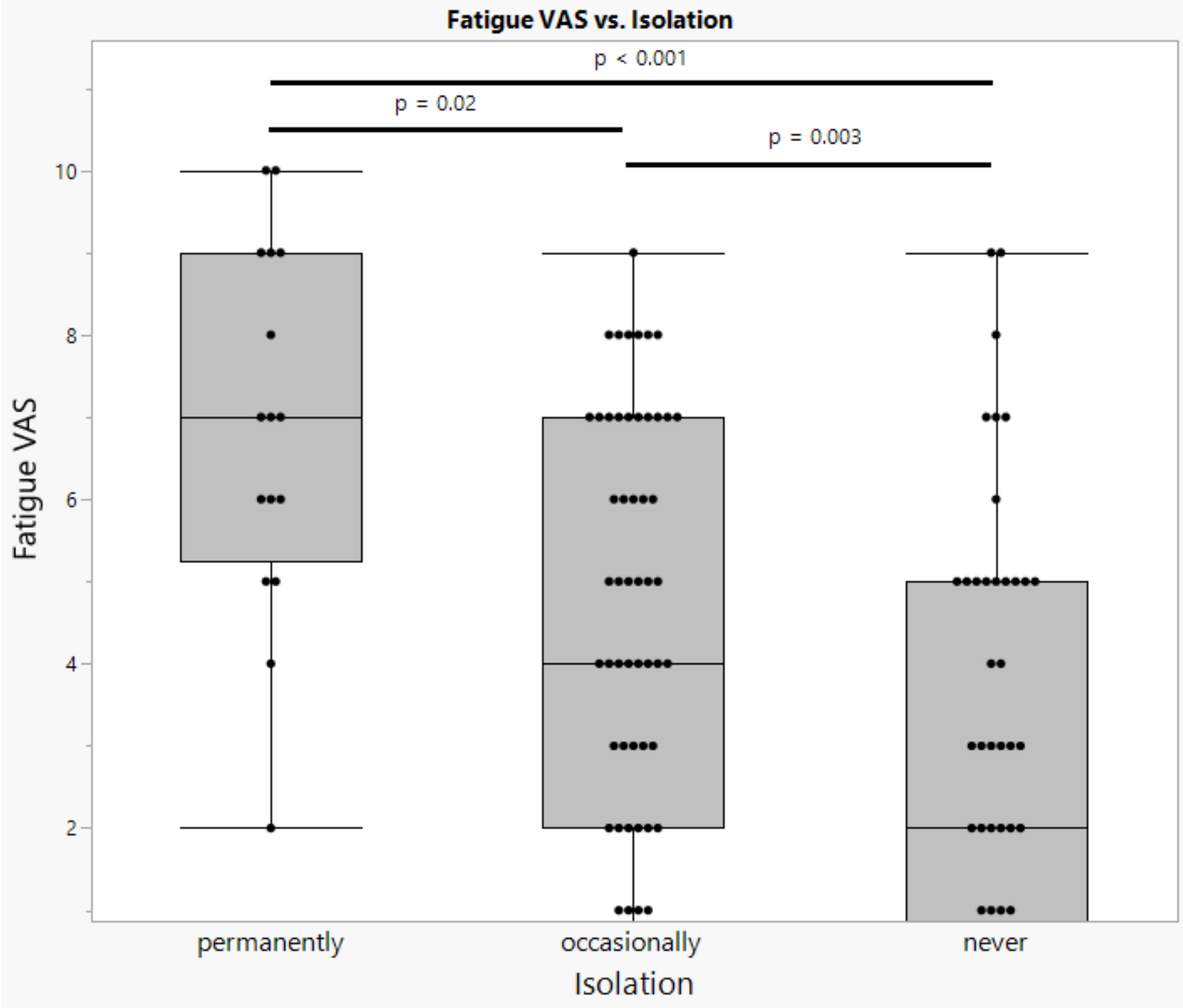

Figure S10

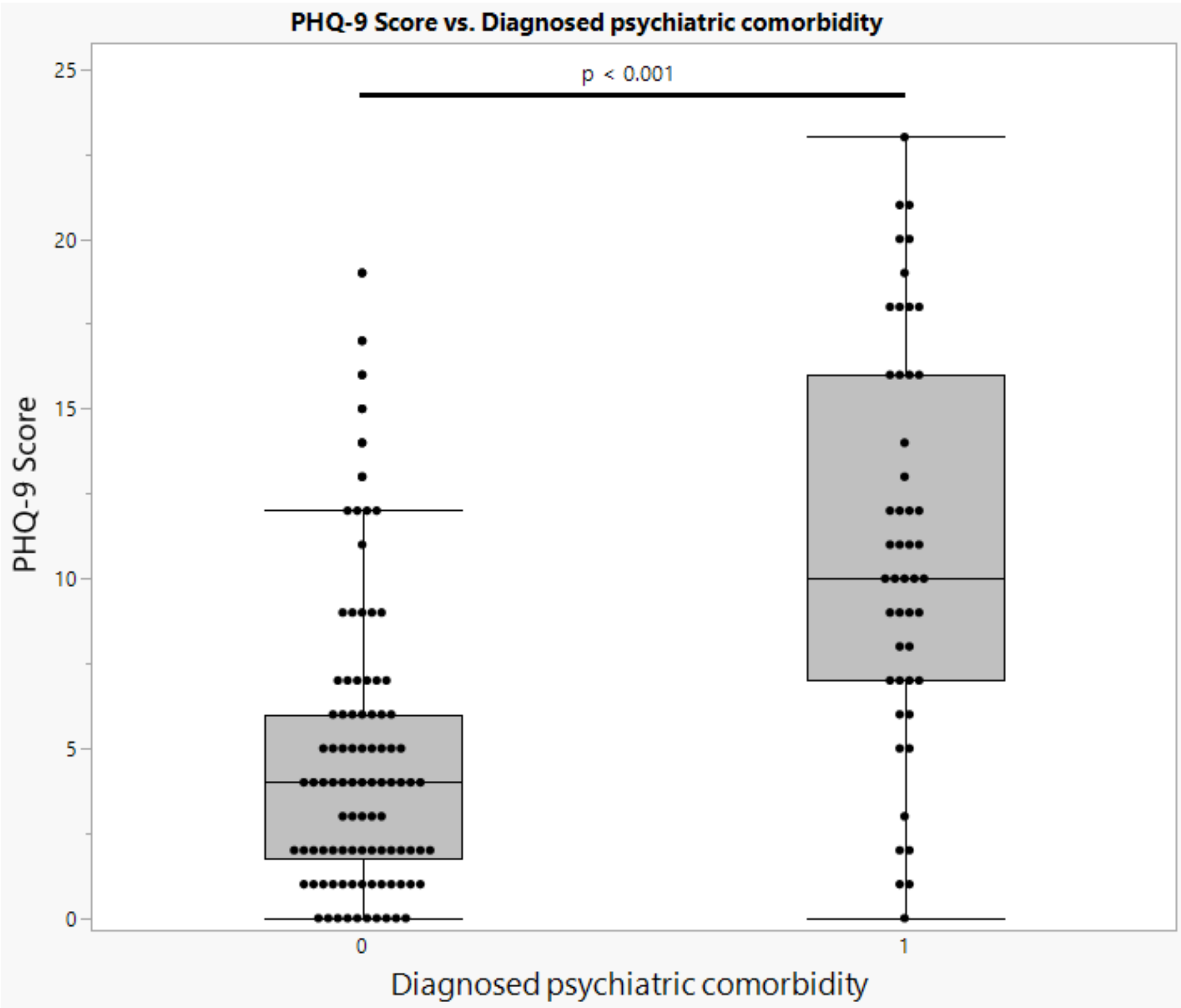

Figure S11

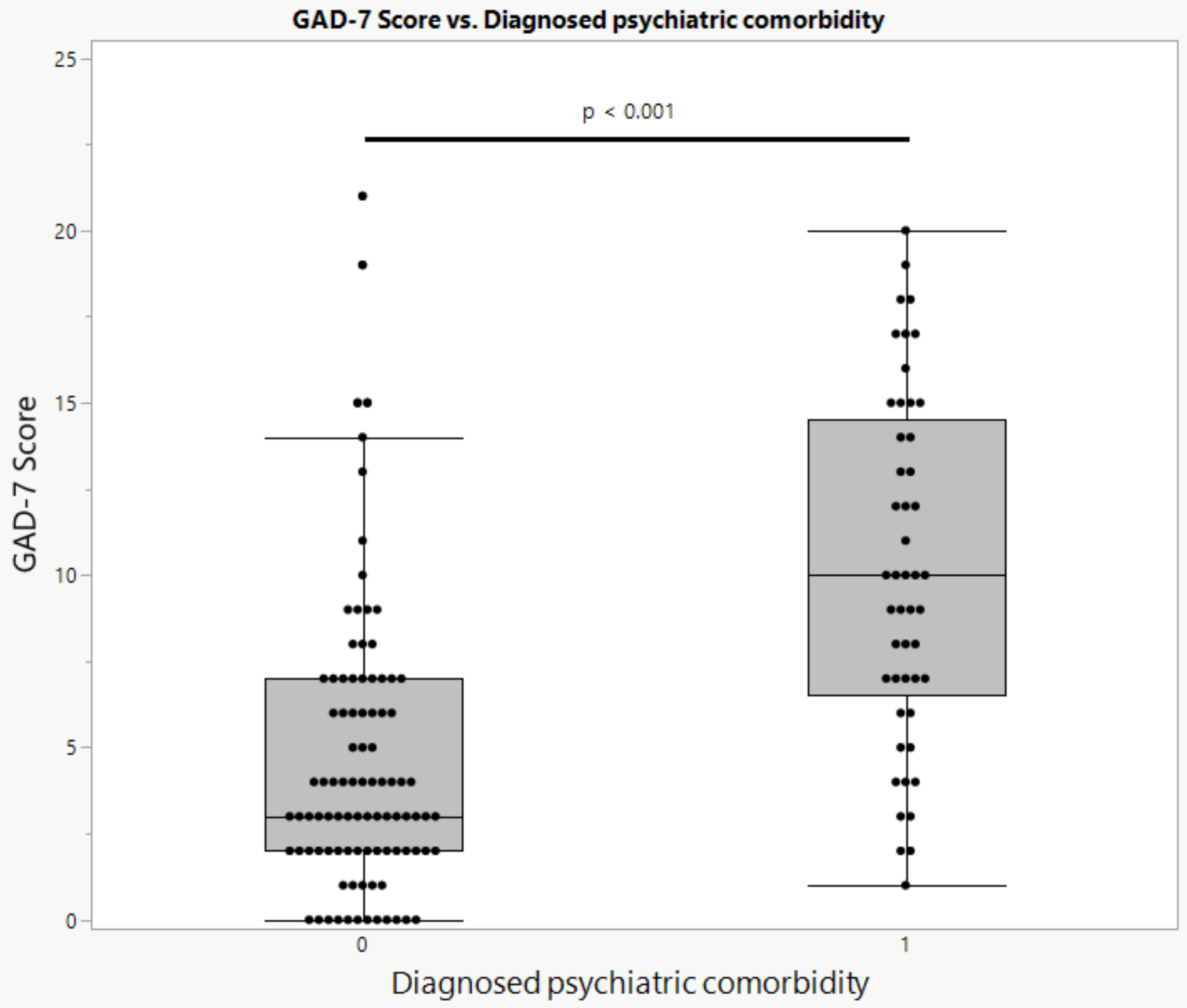

Figure S12

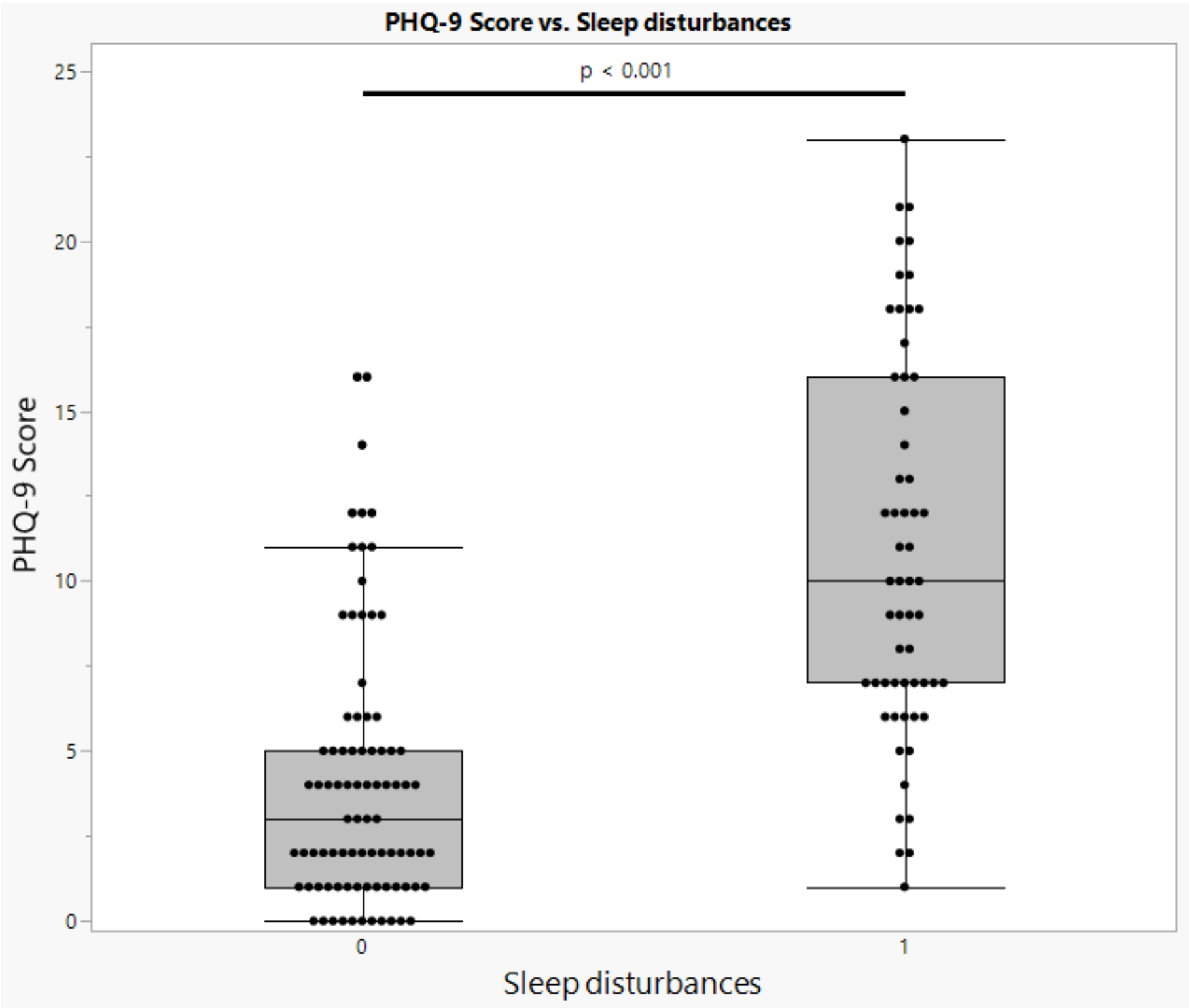

Figure S13

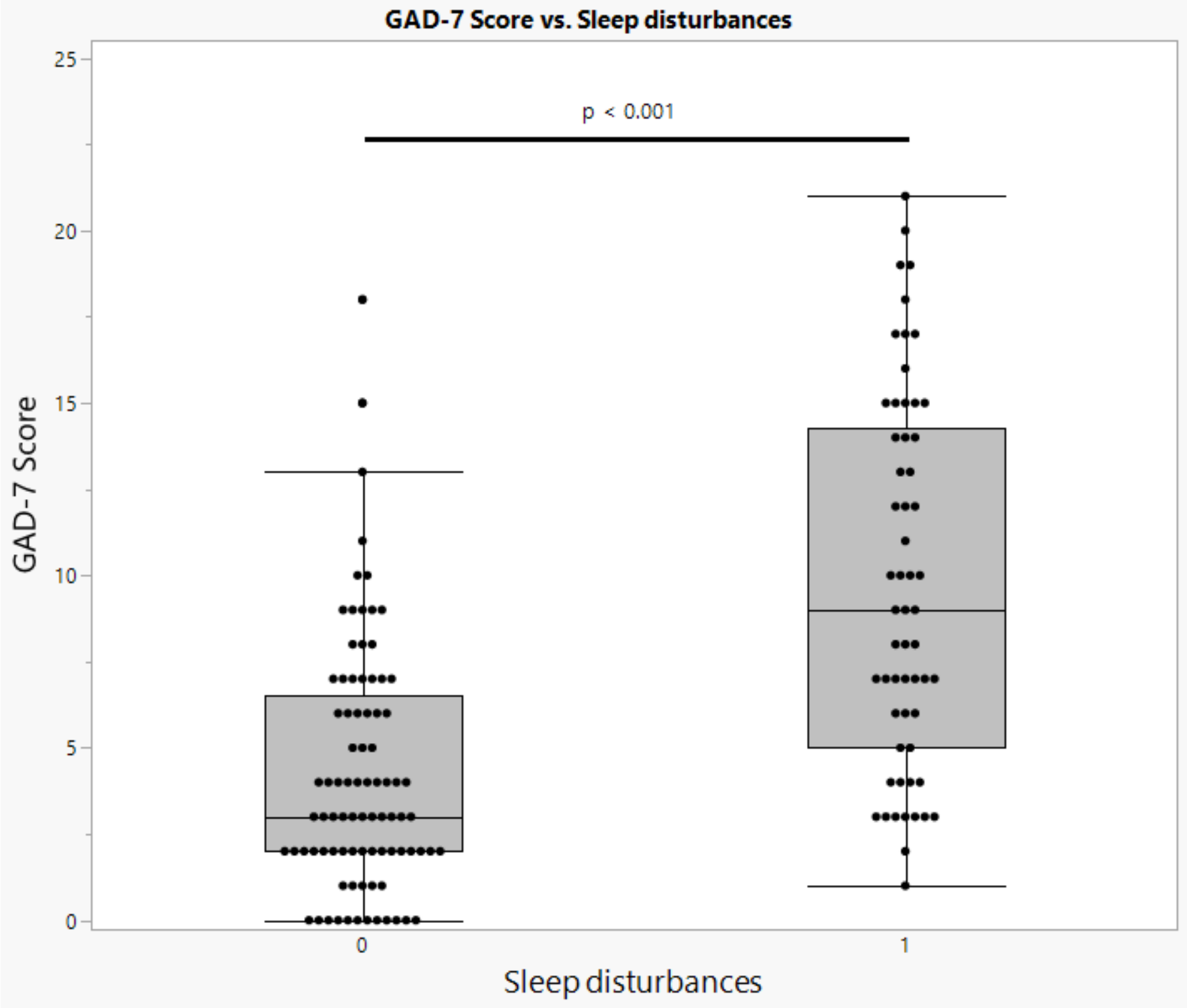

Figure S14

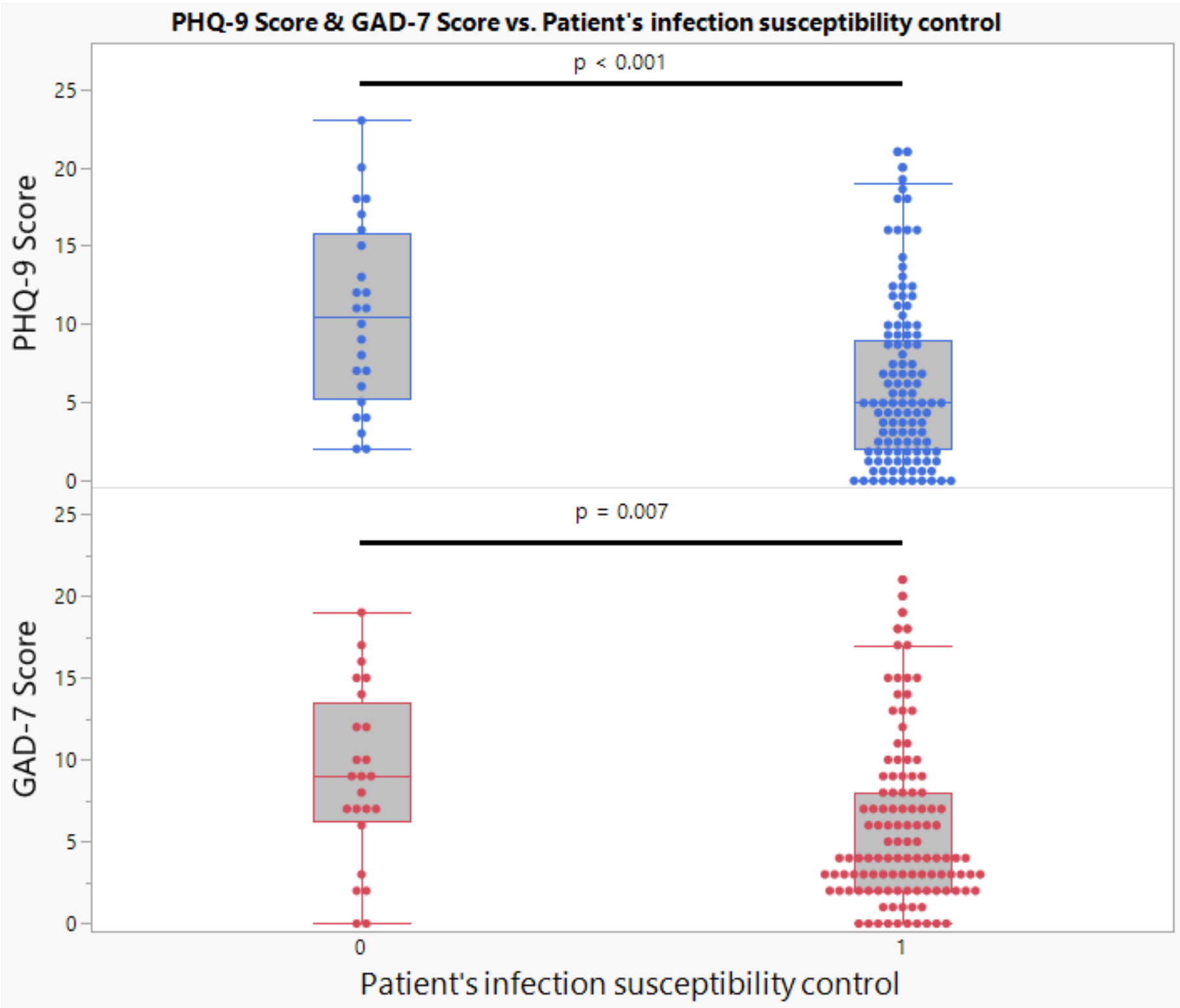

Figure S15

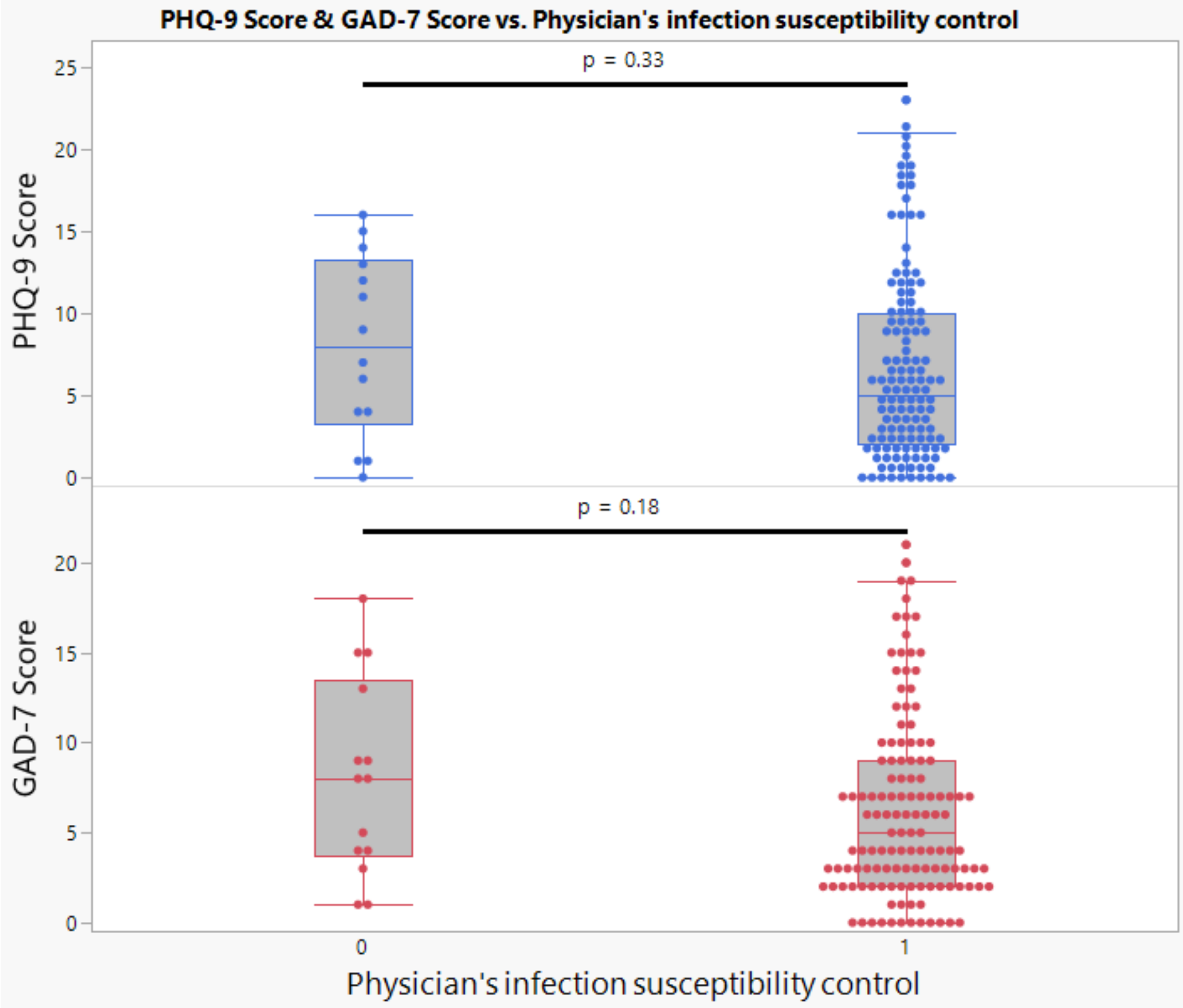
PHQ-9 Score & GAD-7 Score vs. Physician's infection susceptibility control
p = 0.33
p = 0.18
PHQ-9 Score
GAD-7 Score
25
20
15
10
5
0
0
1
Physician's infection susceptibility control
